# In Vivo Skin Regeneration and Wound Healing Using Cell Micro-Transplantation

**DOI:** 10.3390/pharmaceutics14091955

**Published:** 2022-09-15

**Authors:** Lucia Nanić, Andrea Cedilak, Nikolina Škrobot Vidaček, Florian Gruber, Miljenko Huzak, Michael Bader, Ivica Rubelj

**Affiliations:** 1 Laboratory for Molecular and Cellular Biology, Division of Molecular Biology, Ruder Boskovic Institute, 10000 Zagreb, Croatia; 2 Department of Dermatology, Medical University of Vienna, 1090 Vienna, Austria; 3 Department of Mathematics, Faculty of Science, University of Zagreb, 10000 Zagreb, Croatia; 4 Max-Delbrück-Center for Molecular Medicine in the Helmholtz Association (MDC), 13125 Berlin, Germany; 5 German Center for Cardiovascular Research (DZHK), Partner Site Berlin, 10785 Berlin, Germany; 6 Charité Universitätsmedizin Berlin, Corporate Member of Freie Universität Berlin and Humboldt-Universität zu Berlin, 10117 Berlin, Germany; 7 Institute for Biology, University of Lübeck, 23562 Lübeck, Germany

**Keywords:** skin, skin aging, senescence, SASP, revitalization, rejuvenation

## Abstract

*Background:* The accumulation of senescent cells in tissues alters tissue homeostasis and affects wound healing. It is also considered to be the main contributing factor to aging. In addition to losing their ability to divide, senescent cells exert detrimental effects on surrounding tissues through their senescence-associated secretory phenotype (SASP). They also affect stem cells and their niche, reducing their capacity to divide which increasingly reduces tissue regenerative capacity over time. The aim of our study was to restore aged skin by increasing the fraction of young cells in vivo using a young cell micro-transplantation technique on Fischer 344 rats. Employing the same technique, we also used wild-type skin fibroblasts and stem cells in order to heal Dominant Dystrophic Epidermolysis Bulosa (DDEB) wounds and skin blistering. *Results:* We demonstrate that implantation of young fibroblasts restores cell density, revitalizes cell proliferation in the dermis and epidermis, rejuvenates collagen I and III matrices, and boosts epidermal stem cell proliferation in rats with advancing age. We were also able to reduce blistering in DDEB rats by transplantation of skin stem cells but not skin fibroblasts. *Conclusions:* Our intervention proves that a local increase of young cells in the dermis changes tissue homeostasis well enough to revitalize the stem cell niche, ensuring overall skin restoration and rejuvenation as well as healing DDEB skin. Our method has great potential for clinical applications in skin aging, as well as for the treatment of various skin diseases.

## 1. Introduction

Cellular senescence has long been considered a mechanism that protects the body from unrestricted growth of damaged cells [1,2] and the development of tumors [3]. Recent studies have shown that it also plays an important role in complex biological processes such as embryonic development [4,5], wound healing [6], and tissue repair [7]. In addition to its important roles across the lifespan, experimental evidence suggests that the accumulation of senescent cells has a major influence on human aging as well [8,9]. Although they lose the ability to divide, senescent cells remain viable and metabolically active but undergo dramatic changes in gene expression [10,11,12]. Among the most important changes in senescent cell gene transcription profiles are the expression of proinflammatory cytokines and chemokines, various growth factors as well as matrix metalloproteinases which together contribute to impaired tissue structure and homeostasis with aging [13,14,15,16]. This altered secretory phenotype in senescence is known as SASP (senescence-associated secretory phenotype) [17]. An important aspect of SASP is the evolutionarily conserved expression profile of many factors between rodents (mice) and humans [17,18], which makes them a good experimental model relevant to humans.

In addition to the changed structure and function of normal tissues [19], SASP provokes an immune system response creating a metabolic profile of inflammation [9]. Lysophosphatidylcholines, the bioactive lipids, were also recently identified as SASP factors that may facilitate immune evasion and low-grade chronic inflammation in skin aging [20]. Importantly, primary senescent cells induce senescence in neighboring normal cells [21]. They also stimulate the growth and development of preneoplasia [14,22,23,24] but at the same time, they do not stimulate the growth of normal epithelial cells [25,26,27].

Aged skin is characterized by decreased numbers of young fibroblasts which reduces the synthesis of collagen and elastin, existing elastin undergoes progressive calcification and degradation and collagen fibers lose their organized structure [28,29]. Changes in the mechanical properties of the skin are the result of the degradation of existing extracellular matrix (ECM) proteins including collagen type I, III, IV, and VII, fibronectin, elastin, and fibrillin due to the activity of matrix metalloproteinase (MMP) enzymes secreted by senescent cells. Increased expression of MMPs [30] also promotes a pro-oxidative environment in aging skin which further disturbs the stem cell pool. Skin stem cells are sensitive to senescence-related changes in their microenvironment so that their regenerative potential progressively decreases leading to attenuation of continuous tissue renewal [31]. Research suggests that stem cells in an old organism showing reduced proliferative potential can be rejuvenated if some positive changes occur in their systemic microenvironment [32].

Skin aging research involving the use of stem cells or young somatic cells has so far been mostly performed on cell cultures or on animal models in which the cells were injected with a medical needle [33,34]. Research in which young cells were transplanted diffusely into the skin at a larger surface and throughout all layers is widely missing. In this paper, we used diffuse cell micro-transplantation in order to treat old or DDEB-affected rat skin in vivo.

In addition to aging, various genetic disorders can disrupt the protective function of the skin. Among the most severe is Epidermolysis bullosa (EB), a heterogeneous group of hereditary skin fragility disorders caused by mutations in 18 different genes resulting in a wide range of pathologies, from mild to severe, and in extreme cases can cause death. These genes code for various skin proteins important for dermal-epidermal connections. One of the best known is Collagen VII (C7) which anchors the basal membrane. Affected animals carry mutation within the major structural (collagenous) domain of C7 which decreases the stability of C7 monomers conferring dominant-negative interference. They have fragile and blister-prone skin showing all major signs of EB in humans [35]. We show that it is possible to improve dermis-epidermis connection and wound healing by increasing the ratio of wild-type young cells in the skin.

Currently, it is widely accepted that the accumulation of senescent cells and an increased level of SASP is a driving force in reduced tissue regeneration capacity, impaired tissue and organ functions, and overall body deterioration, the well-known hallmarks of aging [36,37,38]. To reduce or eliminate the harmful effects of senescent or dysfunctional cells in aged or wounded skin, we introduced a method for cell micro-transplantation by which we were able to change local tissue composition, using the rat as a model organism.

## 2. Materials and Methods

### 2.1. Animals

Fisher 344 rats—an isogenic rat line was used for cell heterotransplantation experiments. Changes in cell number and density were followed in the skin of untreated rats of increasing ages (3, 10, 17, and 24-month-old animals, *n* = 3). For cell transplantation experiments, animals of increasing age were used (6, 12, 18, and 24 months; *n* = 6) and samples were explanted after 4–6 weeks.

For the long-term experiments, 12-month-old rats were used (*n* = 3) at the time of implantation, the first explantation was carried out at 6 weeks, and the second 10 months following implantation.

Epidermolysis bullosa experiments were carried out on a DDEB rat strain (Sprague Dawley rats originally housed at the Max Delbruck Center for Molecular Medicine, Berlin; DDEB phenotype had developed due to a spontaneous mutation [35]; 3–4 samples per analysis).

### 2.2. Primary Cell Culture

Newborn Fisher 344 rats, up to 5 days old, were sacrificed, their skin explants were collected and digested in 0.25% trypsin solution in order to establish the primary fibroblast culture. Isolated cells were maintained in Dulbecco's modified minimal medium (DMEM, Sigma, St. Louis, MO, USA) supplemented with 10% fetal bovine serum (FBS, Gibco, Gaithersburg, MD, USA) and 2.5× Penicillin/Streptomycin/Amphotericin B (PSA, Sigma) and subcultured at 90% confluency. For experiments for Epidermolysis bullosa, epidermal stem cells were isolated from newborn wild-type Sprague Dawley rat skin as described by Forni et al. [39] and maintained in Keratinocyte-SFM media (K-SFM, Gibco). Upon isolation, stem cells were positively identified by antibody anti-alpha 6 integrin.

All experimental protocols were carried out according to the ILAR Guide for the Care and Use of Laboratory Animals, the Directive on the protection of animals used for scientific purposes (2010/63/EU), and the Croatian Animal Protection Act (Official Gazette 135/06, 37/13 and 125/13) and in their implementation the principles of 3R were strictly followed. The personnel involved in the conduct of experiments on animals possess a valid license (FELASA category C equivalent).

### 2.3. Micro-Transplantation Treatment

Carbocyanine dye DiI (1,1-dioctadecyl-3,3,3,3-tetramethylindolecarbocyanine perchlorate) was used to label fibroblast cell membranes in order to identify implanted cells in the skin. DiI was added to the DMEM with 10% FBS at a final concentration of 5 µM and the cells were incubated in the dark for 20 min at 37 °C, resuspended, and washed 3 times in DMEM with 10% FBS. Animals were anesthetized with a mixture of 5% isoflurane and oxygen in an induction chamber (SomnoSuite, Kent Scientific, Torrington, CT, USA). At least one week prior to the implantation, several 1 cm^2^ fields were permanently marked in the dorsal rat skin using a tattoo machine (iStar 3000, MEI-CHA, Lake Forest, CA, USA) with 1-prong needle. Micro-transplantations were carried out using the same machine but with 11-prong needles. In each treatment 8 × 10^6^ cells in 150 µL serum-free DMEM were implanted into a 1 cm^2^ dorsal skin field. As a mock mechanical control, only a serum-free DMEM was used. Unless stated otherwise, rats were left to heal for 4–6 weeks prior to skin sampling. The same procedure was employed to implant skin fibroblasts or epidermal stem cells into the skin of DDEB model rats [35], with an exception of using K-SFM media instead of DMEM. Three different biopsy samples were collected from each anesthetized rat; untreated tissue (negative control; NC), mechanically treated tissue (mechanical control; MC), and tissue treated with young cells (TT). Tissue was fixated in neutral buffered 10% formalin (10% NBF, Sigma). One part of each sample was fixated at room temperature overnight and embedded in paraffin. The other part was fixated for two hours at 4 °C, cryoprotected first in 5% sucrose overnight at 4 °C and then in 30% sucrose overnight at 4 °C. It was then mounted in freezing medium O.C.T. (Sakura, Tokyo, Japan) and stored at −80 °C.

### 2.4. Skin Tissue Analysis

DiI detection. Frozen tissue sections (7 µm) were stained with Hoechst 33342 (Invitrogen, San Diego, CA, USA) for 20 min at room temperature.

Hematoxylin and eosin staining. Paraffin-embedded sections (4 µm) were dewaxed, rehydrated, and stained with hematoxylin (Merck, Rahway, NJ, USA) for 10 min at room temperature, washed with tap water, and counterstained with eosin (Merck) for 5 min.

Immunofluorescence. Paraffin-embedded sections (4 µm) were dewaxed, rehydrated and antigen retrieval was carried out by heat-induced antigen retrieval treatment in 10 mM citrate buffer pH 6.0 (Dako, Fargo, ND, USA). Samples were incubated in 10% normal serum (rabbit or mouse) and 1% BSA in PBS for 1 h at room temperature, followed by incubation with the primary antibody in 1% BSA in PBS overnight at 4 °C (Rabbit Anti-Ki67, 5 µg/mL; Rabbit Anti-Collagen I, 5 µg/mL; Mouse Anti-Collagen III,1:600; Abcam, Cambridge, MA, United States). The sections were then washed and incubated with the secondary antibody (Anti-Rabbit IgG Alexa Fluor 488, 1:500; Anti-Mouse IgG Alexa Fluor 647, 1:500; Abcam) in 10% normal serum and 1% BSA in PBS for 1 h at room temperature in the dark. Samples were counterstained with Hoechst 33342, washed and embedded in a mounting medium (Permafluor, Thermo Scientific, Boston, MA, USA).

### 2.5. Cell Distance Measurement

To measure the average cell distance in the skin, three hematoxylin-eosin stained tissue sections for each field (NC, MC, and TT) were used per animal. Four fields sized 100 µm × 100 µm from three separate sections per animal were analyzed. Each age group consisted of three animals. Average cell distance was calculated according to the formula:

$$\mathrm{Average}\;\mathrm{cell}\;\mathrm{distance}=\frac{100\;\mu m}{\sqrt{\mathrm{Number}\;\mathrm{of}\;\mathrm{cells}}}\cdot \frac{1+\sqrt{2}}{2}$$

Measurement was performed using ImageJ software (NIH). A detailed mathematical description of the measurement and comprehensive statistical analysis are presented in the Supplementary Materials.

### 2.6. Immunofluorescence Analysis

Immunofluorescence signal readings for collagen I and collagen III were obtained by ImageJ software and analyzed using the Corrected Total Cell Fluorescence (CTCF) calculation method [40] according to the formula:

CTCF = integrated density − (area of selected field × mean fluorescence of background readings).

The analysis was performed for three fields of each section, and three sections were processed for each animal. Each group consisted of three animals.

### 2.7. Statistical Analysis

For analyzing dependency of a measured variable Y (or its transforms) with respect to variable Age in months (x), we used a linear regression model with Age in months as a regressor. Significance of the model, model adequacy, and lack of fit were tested by usual F-tests. We compared laws of Y with respect to the grouping factor (with classes NC, MC, and TT) by methods of one-factor ANOVA. All model parameters were estimated by the ordinary or weighted least square methods. The main assumptions for inference purposes were that Y (or it transforms wherever appropriated) had normal distribution laws in all analyzed subsamples. Normality was tested by Shapiro–Wilk and Lilliefors variant of Kolmogorov-Smirnov tests, and graphically by use of normal probability plots. Statistical analysis was performed by Statistica (ver. 13, TIBCO Software Inc., Palo Alto, CA, USA) and MATLAB (R2010b, The MathWorks, Inc., Natick, MA, USA) software. Comprehensive statistical analysis is presented in the Supplementary Materials.

Graphs were generated in Statistica. Error bars represent mean ± 0.95 confidence intervals. Significant differences are indicated by asterisks (* *p* < 0.05; ** *p* < 0.01; *** *p* < 0.001; **** *p* < 0.0001).

## 3. Results

### 3.1. Implantation of Young Fibroblasts Restores Cell Density in Aged Rat Skin

**Figure 1:**
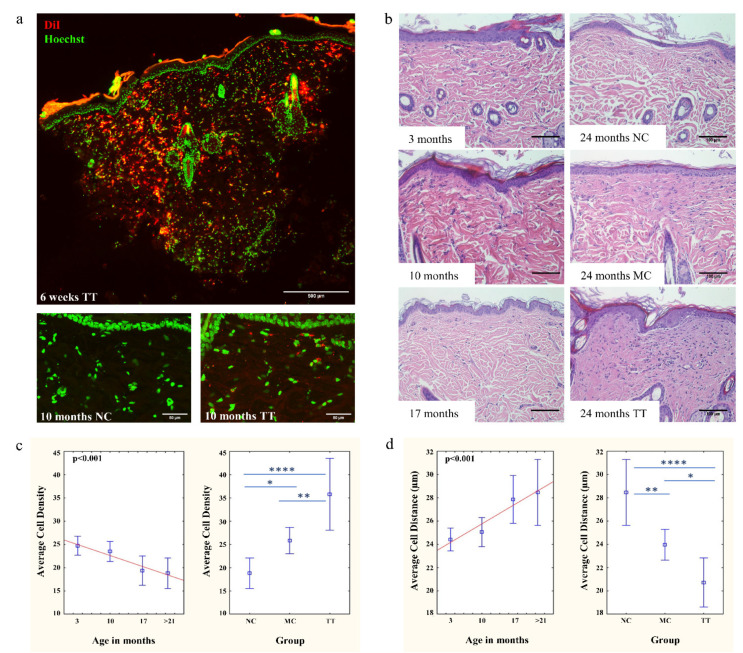
Young fibroblasts are efficiently implanted into aged rat skin. (**a**) Successful implantation of fibroblasts (red) in skin of 12-month-old animal 6 weeks following treatment (6 weeks TT); cell nuclei depicted in green. Scale bar is 500 µm. Ten months following treatment, cells are stably implanted and viable (10 months TT and NC); scale bar is 50 µm. (**b**) Aging dynamics in skin were followed by hematoxylin/eosin staining in 3, 10, and 17-month-old rat skin. H&E of 24-month-old rat skin shows non-treated (NC), mechanically treated (MC), and tissue treated with young cells (TT) from same animal; scale bar is 100 µm. Left graphs represent (**c**) average cell density and (**d**) average cell distance in untreated skin. Right graphs represent changes in cell density or distance in old animals following micro-transplantation procedure. *n* = 3 per group; error bars represent mean ± 0.95 confidence intervals; * *p* < 0.05; ** *p* < 0.01; **** *p* < 0.0001.

For the optimization of the micro-transplantation procedure, experiments were carried out using DiI labeled young neonatal skin fibroblasts isolated from isogenic Fischer 344 rats. Cells were transplanted into the dorsal skin of 12-month-old animals. Our results showed that the most successful implantation was achieved by a single micro-transplantation treatment using 8 × 10^6^ cells per 1 cm^2^ field (Figure S15). Six weeks following treatment, implantation efficiency for newly embedded cells ranged from ~14% to ~50.6%, with an average of ~33.11% (Figure 1a). Importantly, a large number of DiI-labeled cells were found throughout the dermis, even deep below the capillary network. Follow-up analysis of the same animal demonstrated that the implanted cells are stable and viable even after 10 months in the skin. However, due to cell divisions, the intensity of DiI fluorescence decreased compared to the first sampling (Figure 1a). To the best of our knowledge, this is the longest period in which DiI-stained cells were followed in vivo.

Further, we analyzed the skin aging dynamics of the Fischer 344 rats. We observed a constant and significant change in skin aging of untreated 3, 10, 17, and 21–24 month (denoted as > 21 months) old rats (Figure 1b). The most remarkable changes were a decrease in cell density (Figure 1c), and consequently, an increase in the average distance between individual cells (Figure 1d), both of which affect cell-to-cell signaling. Average cell density decreased from ~24.69 cells in three-month-old animals to ~18.78 cells in 21–24 months old animals. These changes demonstrate significant alterations in skin homeostasis and architecture with advancing age (Figure 1c).

Following the analysis of skin aging dynamics of Fischer 344 rats and the successful optimization of our newly developed micro-transplantation procedure, we were able to test the hypothesis that the accumulation of senescent cells is one of the main driving forces of aging. All transplantation experiments were carried out on 21–24 month (denoted as >21 months) old rats using 8 × 10^6^ young DiI labeled cells per 1 cm^2^ marked dorsal area. As a mock mechanical control, only cell medium was used in the area of the same size. Skin samples outside of marked areas were also collected as untreated controls. The implantation of young cells changed aged skin homeostasis, resulting in a significant increase in cell density and a more compact extracellular matrix (Figure 1b). Of particular importance is that following the micro-transplantation treatment, the average number of cells increased to 35.75 which is a >90% increase when compared to untreated tissue (18.78 cells), while mechanical control, as expected, showed a moderate increase to 25.81 cells (Figure 1c). Consequently, the average distance between individual cells decreased from 28.4 µm in untreated tissues to 20.7 µm in tissues of the same animals following the treatment (Figure 1d).

### 3.2. Revitalization of Cell Proliferation in Epidermis and Dermis

**Figure 2:**
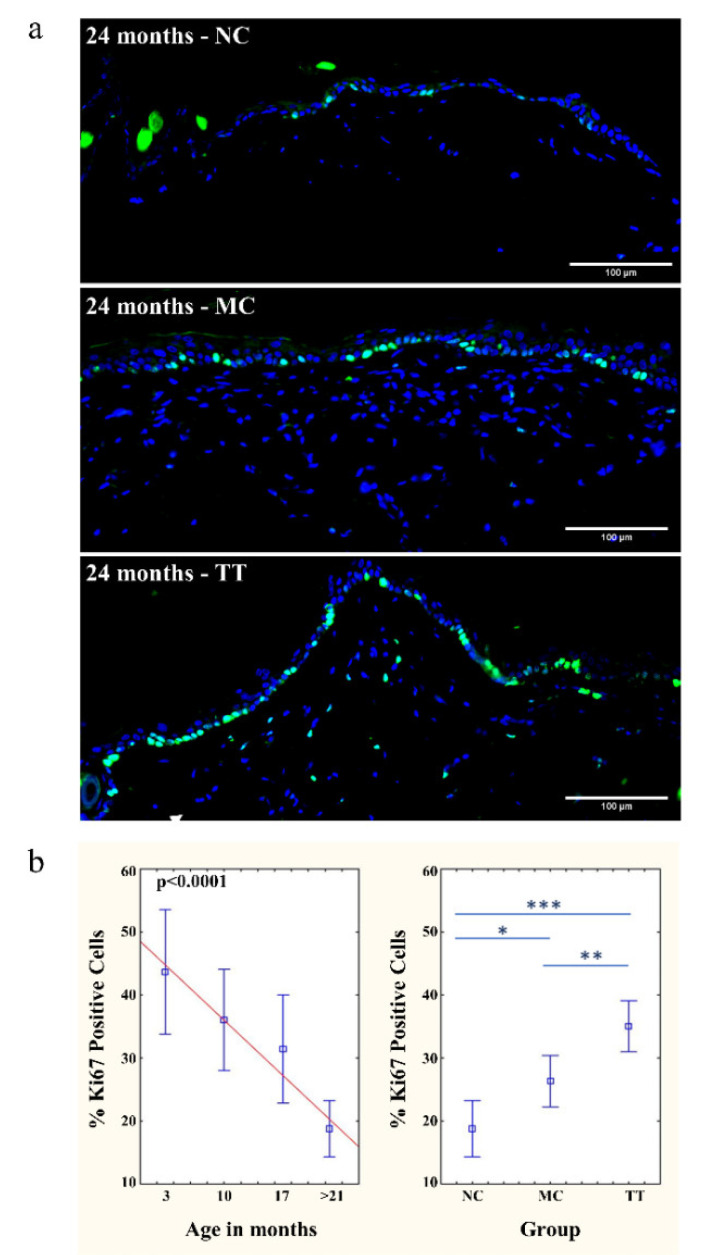
Implanted young fibroblasts have positive effect on the stem cell niche. (**a**) Dynamics of changes in the epidermal cell proliferation status with aging and following micro-transplantation procedure were monitored by Ki67 marker (green). Cell proliferation status is shown in untreated skin (NC), mechanically treated skin (MC), and skin treated with young cells (TT). All samples are from the same 24-month-old animal. Scale bar is 100 µm. (**b**) Graphs represent the percentage of Ki67 positive cells on the basal membrane of the epidermis in the skin of untreated rats (3, 10, 17, and >21 months, NC; left), and changes in proliferation status of >21 months old animals following micro-transplantation procedure (NC, MC, TT; right). *n* = 3 per group; error bars represent mean ± 0.95 confidence intervals; * *p* < 0.05; ** *p* < 0.01; *** *p* < 0.001.

Following the micro-transplantation of young cells into the dorsal skin of 21–24 month (denoted as >21 months) old rats, we also investigated cell proliferation in the epidermis and dermis. Analysis using the Ki67 proliferation marker revealed that most of the Ki67 signals were located on the basement membrane of the epidermis, more specifically in the epidermal stem cell niche (Figure 2a).

The proliferative status of the epidermal stem and cycling cells in untreated skin showed a continuous and significant decrease with aging. The skin of three-month-old animals showed an average of 43.66% Ki67-positive cells at the basement membrane. With aging, proliferative status declines, and in the epidermis of >21-month-old rats averages at 18.79%. (Figure 2b). Following implantation of young cells, the proliferative status of aged tissues improved significantly, resulting in an increase to an average of 35.04% Ki67 positive cells which is equivalent to the proliferative status of 10-month-old animals and is almost twice as high compared to the control untreated tissue samples. Mechanical treatment only moderately improved aged tissue proliferative status to 26.35% (Figure 2b).

In addition to the epidermis, positive differences in the proliferative status of dermal cells were also observed following cell micro-transplantation. As expected, this increase occurs at a much lower frequency than in the stem cell niche, but in comparison, cell divisions were not observed in the dermis of untreated tissue (Figure 2a).

### 3.3. Young Fibroblast Micro-Transplantation Rejuvenates Collagen I and III Matrix in Aged Skin

**Figure 3:**
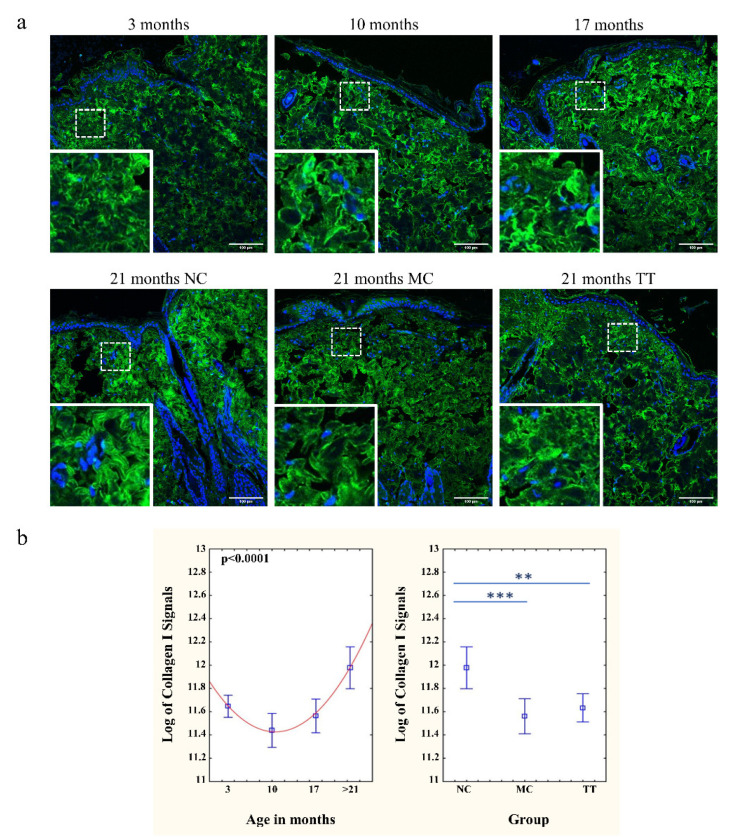
Implanted young fibroblasts change collagen I immunofluorescence pattern in aged skin. (**a**) Collagen I (green) in the untreated skin of rats of different ages (3, 10, 17 months) and in the untreated skin (NC), mechanically treated skin (MC), and skin treated with young cells (TT) of the same 21-month-old rat. The bottom left corner of each picture presents an enlarged selected area. Scale bar is 100 µm. (**b**) Graphs represent CTCF for collagen I immunofluorescence signals in the skin of untreated rats (3, 10, 17, and >21 months, NC; left). Changes in collagen I signals of >21 months old animals following micro-transplantation procedure (NC, MC, TT; right). *n* = 3 per group; error bars represent mean ± 0.95 confidence intervals; ** *p* < 0.01; *** *p* < 0.001.

**Figure 4:**
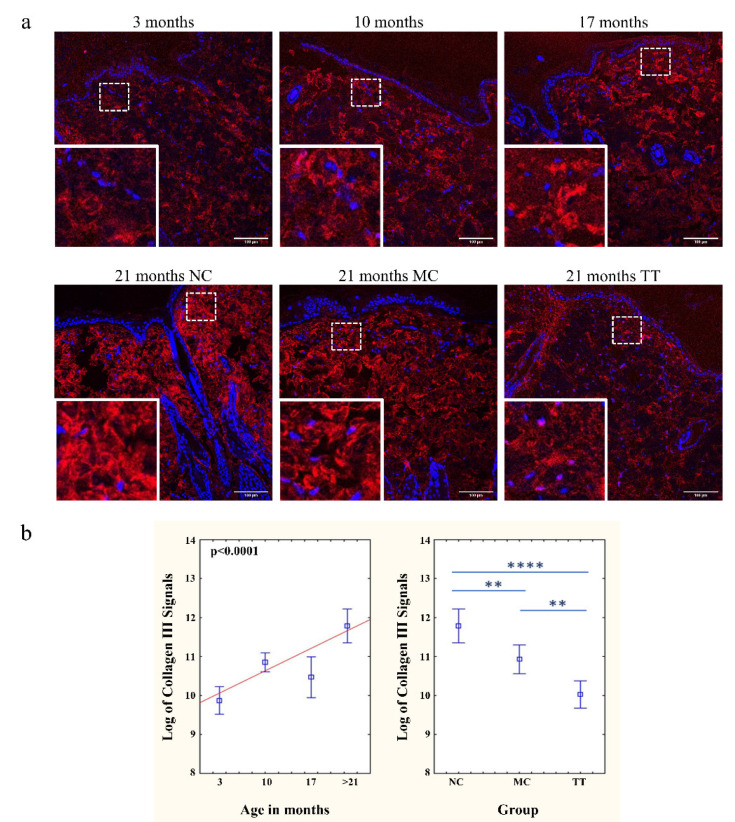
Implanted young fibroblasts change collagen III immunofluorescence pattern in aged skin. (**a**) Collagen III (red) in the untreated skin of rats of different ages (3, 10, 17 months) and in the untreated skin (NC), mechanically treated skin (MC), and skin treated with young cells (TT) of the same 21-month-old rat. The bottom left corner of each picture presents an enlarged selected area. Scale bar is 100 µm. (**b**) Graphs represent CTCF for collagen III immunofluorescence signals in the skin of untreated rats (3, 10, 17, and >21 months, NC; left). Changes in collagen III signals of >21 months old animals following micro-transplantation procedure (NC, MC, TT; right). *n* = 3 per group; error bars represent mean ± 0.95 confidence intervals; ** *p* < 0.01; **** *p* < 0.0001.

The analysis of aging dynamics in dermal ultrastructure revealed that the skin sections of untreated 3, 10, and 17-month-old rats show a lower intensity of immunofluorescence signals for collagen I and III than that of >21-month-old rats (Figure 3a and Figure 4a). As described in the literature the collagens of younger animals are thinner and more properly organized into bundles. In old animals, collagen bundles exhibit an unfolded, scattered structure and also an increased density which reflects a decrease in the spaces between individual bundles [41,42,43,44]. The implantation of young cells into the skin of 21–24 month (denoted as >21 months) old rats initiated a change in dermal ultrastructure and resulted in collagen I and III bundles resembling collagens of 3-month-old rats. The fluorescence intensity of both collagens, following the treatment, had a pattern similar to young skin. In contrast, control aged skin showed a much stronger fluorescence intensity than other samples (Figure 3b and Figure 4b).

### 3.4. Young Fibroblast Micro-Transplantation Boosts Epidermal Stem Cell Proliferation in Rats with Advancing Age

**Figure 5:**
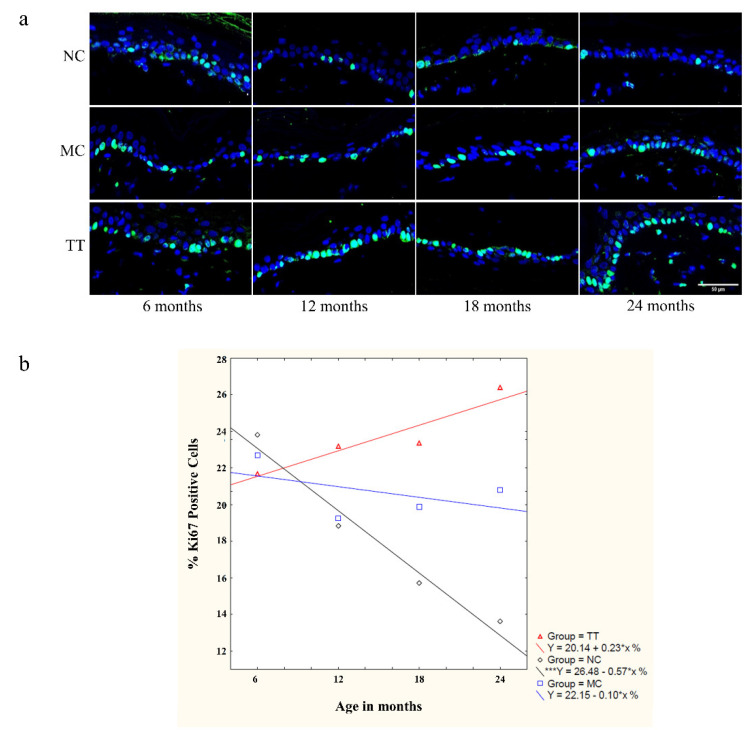
The positive effect of implanted young fibroblasts on stem cell niche is stronger with advancing age. (**a**) Cell proliferation status in rats aged 6, 12, 18, and 24 months (NC-untreated control; MC-mechanical treatment control; TT-young cells implantation treatment). Ki67 positive stem and cycling cells (green) on the basement membrane of the epidermis. Scale bar is 50 µm. (**b**) The graph represents the percentage of Ki67 positive cells on the basal membrane of the epidermis with advancing age for untreated tissue (black diamond), mechanical treatment (blue square), and tissue treated with young fibroblasts (red triangles). *n* = 6 for 6, 12, and 18 months; *n* = 5 for 24-month-old animals; error bars represent mean ± 0.95 confidence intervals; *** *p* < 0.001.

Upon micro-transplantation of young fibroblasts into the dermis of 21–24-month-old rats, we observed robust recovery of epidermal cycling cell and stem cell dividing intensity. Therefore, we wanted to analyze such influence in different age groups, as well. Implantations were carried out by single micro-transplantation treatment using 8 × 10^6^ cells per 1 cm^2^ field of dorsal skin in 6, 12, 18, and 24-month-old Fischer 344 rats. Four to six weeks following implantation, skin samples were collected for analysis. The proliferative status of the epidermal stem and cycling cells was analyzed using the Ki67 proliferation marker. Apparently, both mechanical treatment and young fibroblast micro-transplantation treatment have little influence on epidermal cycling/stem cell proliferation in 6-month-old animals. With advancing age, untreated tissues show a progressive and significant decline in cell proliferation dropping from 23.81% to 13.61% at the age of 6 and 24 months, respectively. We observed a strong positive effect in tissues treated with young dermal fibroblasts, reaching 26.41% in the 24-month-old group, almost doubling the values of untreated controls, and at the same time exceeding the 6-month-old untreated controls. The influence of mechanical treatment moderately increases the percentage of Ki67- positive stem/cycling cells to 20.81% (Figure 5).

### 3.5. Implantation of Wild-Type Epidermal Stem Cells into DDEB Rat Skin Reduces Skin Blistering

**Figure 6:**
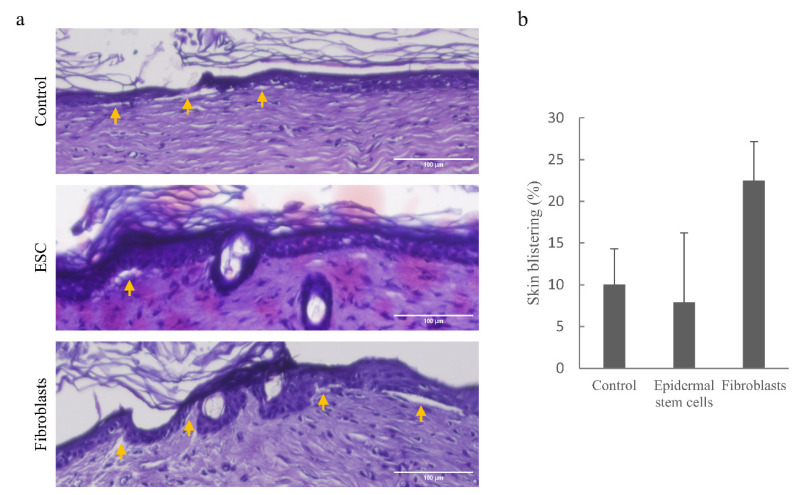
Implantation of wild-type epidermal stem cells or fibroblasts into DDEB rat skin. (**a**) Histological analysis of DDEB rat skin shows skin blistering in control tissue and treated tissues following implantation of wild-type epidermal stem cells (ESC) and fibroblasts. Yellow arrows point to skin blisters on the dermal-epidermal basement membrane. Scale bar is 100 µm. (**b**) The graph represents the percentage of skin blistering prior to and following the treatment. Error bars represent mean ± standard deviation for 3–4 analyzed slides per sample.

In order to restore normal skin morphology, we isolated fibroblasts and epidermal stem cells from newborn wild-type Sprague Dawley rat skin and transplanted them into the skin of the DDEB rat model [35]. Implantations were carried out by single micro-transplantation treatment using 8 × 10^6^ cells per 1 cm^2^ field of dorsal skin. Three months following implantation, skin samples were collected for analysis. In order to determine the percentage of skin blistering in each sample, we measured the blister-affected parts and compared them to the total length of the dermal-epidermal basement membrane of each cross-section. Our results show that blistering in the DDEB skin decreases following ESC implantation by about 21%. Implantation of skin fibroblasts was not beneficial, on the contrary, blistering actually increased following the treatment (Figure 6).

## 4. Discussion

In this study, we have developed a new method of diffuse implantation of cells into skin tissue in order to change the ratio of young cells in the dermis. In heterotransplantation experiments, where young DiI-labeled fibroblasts were implanted into an adult rat skin, we demonstrated the efficiency of the method (14–50.6%; average 33.11%) high enough to impact tissue homeostasis (Figure 1a). Importantly, DiI-labeled cells were found in all skin layers, even deep below the capillary network. This confirms the advantages of this method, such as bypassing the dermal keratinocytes barrier and the hydrophobic layer of the epidermis, directly reaching the desired skin dermal layer.

The micro-transplantation procedure proved to be reliable and reproducible in our hands and it enabled us to test the hypothesis that the accumulation of senescent cells is one of the main causes of pathophysiological changes in tissues and organs that occur with aging [22,25]. Cell micro-transplantation causes mild mechanical damage to the skin which activates wound healing mechanisms as well as immune response. This triggers skin quiescent cells to start dividing and eliminates damaged cells and tissue. During this process the number of dividing cells increases, both due to young implanted cells and endogenous normal cells that take part in the healing process. As reported previously, endogenous senescent cells are unable to divide and they form tissue poorly [45].

By using the micro-transplantation procedure, we demonstrated that the introduction of young cells into the dermis of aged rats locally reverses the process of aging, resulting in a significant increase in the total number of cells in the dermis, recovered epidermal proliferative capacity, and compact collagen matrix in contrast to untreated skin of the same animals. On average, the number of cells in treated aged tissue even exceeds the number of cells in the tissues of three-month-old animals (Figure 1b,c). In all experiments, mechanically treated tissues show moderate improvements. Our results regarding epidermal proliferative capacity corroborate previous research showing that skin stem and cycling cells are sensitive to age-related changes in surrounding tissues which reduce their regenerative potential [31] and that this process is reversible [46]. Following implantation, the proliferative status of the epidermal stem/cycling cells in old skin increased significantly (Figure 2) indicating changes toward young extracellular signaling and SASP attenuation. These effects, both increase in cell density and cell viability, rise significantly with advancing age (Figure 5).

With respect to the extracellular matrix, we observed that skin collagen fibers in younger animals are thinner and more properly organized, whereas, in older animals, collagen bundles exhibit a disordered, scattered structure (Figure 3 and Figure 4) consistent with the literature data [41,42,43,44]. The much stronger intensity of the collagen immunofluorescence signals in the skin of old rats indicates their altered organization, i.e., the scattering of the bundles themselves. Old collagen bundles also occupy a larger area, since the density of cells in the old dermis is significantly lower. However, the skin tissue of old rats following the micro-transplantation of young cells does not resemble untreated tissue but rather looks like the skin of young animals regarding the appearance of collagen bundles.

Besides skin aging, cell micro-transplantation proves to be a promising method for skin wound healing and treatment of diseases like Epidermolysis bullosa. Since type VII collagen protein is produced by both fibroblasts and keratinocytes to form anchoring fibrils that attach the dermis to the basement membrane in vitro [47], we decided to perform such an experiment in our experimental system in vivo. Micro-transplantation of ESC reduced skin blistering while fibroblasts demonstrated the opposite effect confirming that the former is the main source of basal membrane collagen VII in rats in vivo. Our findings suggest that in vitro experiments may not adequately represent processes in the skin in vivo due to the multiple factors involved in skin healing following cell micro-transplantation.

In brief, we have demonstrated: (i) micro-transplantation treatment is suitable for implantation of young cells into an aged skin, (ii) relative reduction of senescent cells in aging tissue attenuates SASP, (iii) if introduced in significant numbers, young cells can alter tissue homeostasis in aged skin and locally reverse the aging process showing accelerated regeneration and rejuvenation, (iv) rejuvenation effects last for at least six weeks, the period of our post-treatment monitoring, and (v) micro-transplantation of ESC can be beneficial in reducing DDEB symptomatology.

Recognizing the potential of our methodology and results for clinical application, we intend to continue future investigation towards methodology improvement and more detailed characterization of the rejuvenation or healing phenotype and mechanisms underlying it.

## Data Availability

The datasets used and/or analyzed during the current study are available from the corresponding author upon reasonable request.

## Supplementary Materials

The following supporting information can be downloaded at: https://www.mdpi.com/article/10.3390/pharmaceutics14091955/s1, Figure S1: Normal probability plots of % Ki67 positive cells subsamples; Figure S2: Normal probability plots of % Ki67 positive cells residuals; Figure S3: Normal probability plots of Number of cells subsamples; Figure S4: Normal probability plots of Number of cells residuals; Figure S5: Normal probability plots of Average cell distance subsamples; Figure S6: Normal probability plots of Average cell distance residuals; Figure S7: Mean plots with error bars of Log Signals of collagen I subsamples; Figure S8: Normal probability plots of Log Signals of collagen I residuals; Figure S9: Mean plots with error bars of Log Signals of collagen III subsamples; Figure S10: Normal probability plots of Log Signals of collagen III residuals; Figure S11: Normal probability plots of % Ki67 positive cells subsamples; Figure S12: Plot of % Ki67 positive cells residuals of fitted full linear models and their normal probability plot; Figure S13: Mean plots of % Ki67 positive cells with error bars and their linear models by groups separately; Figure S14: Normal probability plots of % Ki67 positive cells subsamples; Figure S15: Normal probability plot of residuals, and mean plots with error bars of Square-root of Dil positive cells Table S1: Descriptive statistics and tests of normality for percentage of Ki67 positive cells; Table S2: ANOVA table for linear regression model fitting of %Ki67 positive cells, group NC; Table S3: Estimates of the parameters of linear regression model for %Ki67 positive cells, group NC; Table S4: ANOVA table of % Ki67 positive cells for groups NC, MC, and TT at *x* = 22.5; Table S5: Comparison of group population means of % Ki67 positive cells for groups NC, MC, and TT at *x* = 22.5, by Tuckey’s HSD and Fisher’s LSD tests; Table S6: Descriptive statistics and tests of normality for Number of cells; Table S7: ANOVA table for linear regression model fitting of Number of cells, group NC; Table S8: Estimates of the parameters of linear regression model for Number of cells, group NC; Table S9: ANOVA table of Number of cells for groups NC, MC, and TT at *x* = 22.5; Table S10: Comparison of group population means of Number of cells for groups NC, MC, and TT at *x* = 22.5, by Tuckey’s HSD and Fisher’s LSD tests; Table S11: Descriptive statistics and tests of normality for Average cell distance; Table S12: ANOVA table for linear regression model fitting of Average cell distance, group NC; Table S13: Estimates of the parameters of linear regression model for Average cell distance, group NC; Table S14: ANOVA table of Average cell distance for groups NC, MC, and TT at *x* = 22.5; Table S15: Comparison of group population means of Average cell distance for groups NC, MC, and TT at *x* = 22.5, by Tuckey’s HSD and Fisher’s LSD tests; Table S16: Descriptive statistics and tests of normality for Log Signals of collagen I; Table S17: ANOVA tables for quadratic regression model fitting of Log Signals of collagen I, group NC; Table S18: Estimates of the parameters of quadratic regression model for Log Signals of collagen I, group NC; Table S19: ANOVA table of Log Signals of collagen I for groups NC, MC, and TT at *x* = 22.5; Table S20: Comparison of group population means of Log Signals of collagen I for groups NC, MC, and TT at *x* = 22.5, by Tuckey’s HSD and Fisher’s LSD tests; Table S21: Descriptive statistics and tests of normality for Log Signals of collagen III; Table S22: ANOVA tables for quadratic regression model fitting of Log Signals of collagen III, group NC; Table S23: Estimates of the parameters of quadratic regression model for Log Signals of collagen III, group NC; Table S24: ANOVA table of Log Signals of collagen III for groups NC, MC, and TT at *x* = 22.5; Table S25: Comparison of group population means of Log Signals of collagen III for groups NC, MC, and TT at *x* = 22.5, by Tuckey’s HSD and Fisher’s LSD tests; Table S26: Descriptive statistics and tests of normality for percentage of Ki67 positive cells; Table S27: ANOVA table for the compound linear regression model fitting of %Ki67 positive cells; Table S28: Estimates of the parameters of the linear regression models for %Ki67 positive cells; Table S29: Descriptive statistics and tests of normality for Sqrt of Dil pos. cells cells; Table S30: ANOVA table of Square-root of Dil positive cells for groups I, II, and III; Table S31: Comparison of group population means of Square-root of Dil positive cells for groups I, II, and III, by Tuckey’s HSD and Fisher’s LSD tests [48,49,50,51,52].
