# Supplementary material for: In Vivo Skin Regeneration and Wound Healing Using Cell Micro-Transplantation
Source: Pharmaceutics. 2022 Sep 15;14(9):1955. doi: 10.3390/pharmaceutics14091955 (PMC9501230; doi:10.3390/pharmaceutics14091955)
Supplement: Supplementary file 1 [file pharmaceutics-14-01955-s001.zip › pharmaceutics-1854362-supplementary.pdf]

Supplement

Statistical Analysis

Statistical analysis was performed by *Statistica* (ver. 13, TIBCO Software Inc.) and *MATLAB* (R2010b, The MathWorks, Inc.) softwares.

Percentage of Ki67 positive cells

Percentage of Ki67 positive cells (Y) was measured independently with respect to variable Age in months (x). The data are classified in three groups: untreated tissue (negative control, NC), mechanically treated tissue (mechanical control, MC) and with young cells treated tissue (TT). In NC class Y was measured several times (n) with respect to Age of x = 3 months, 10 months, 17 months and between 21 and 24 months ($x = 21 - 24$); in MC and TT classes Y was measured several times only for Age of $x = 21 - 24$ months. For the purpose of regression analysis, we approximated Age x to be equal to 22.5 months for all data with Age x between 21 and 24 months. For all data subsamples, we tested normality by Lilliefors variant of Kolmogorov-Smirnov test ([4], Test 7a, pp. 142-143), and by Shapiro-Wilk test [3]. Descriptive statistics of Y together with the normality test results for the subsamples are presented in Table S1. Normal probability plots of the subsamples are in Figure S1.

Table S1: Descriptive statistics and tests of normality for percentage of Ki67 positive cells

Group	Age in months (x)	No.of cases (n)	Mean ($\bar{Y}$)	Std. dev. (σ)	Std. error of mean	Lillie L	test pv	Shapiro -Wilk W	test pv
NC	3	12	0.4366	0.1551	0.0448	0.2192	< 0.15	0.9118	0.2248
NC	10	11	0.3605	0.1195	0.0361	0.1960	> 0.20	0.9137	0.2692
NC	17	11	0.3145	0.1273	0.0384	0.1283	> 0.20	0.9728	0.9130
NC	22.5	22	0.1879	0.1008	0.0215	0.2199	< 0.01	0.8912	0.0199

MC	22.5	21	0.2635	0.0891	0.0194	0.1454	> 0.20	0.9434	0.2536
TT	22.5	23	0.3504	0.0936	0.0195	0.0941	> 0.20	0.9880	0.9907

Figure S2: Normal probability plots of % Ki67 positive cells subsamples

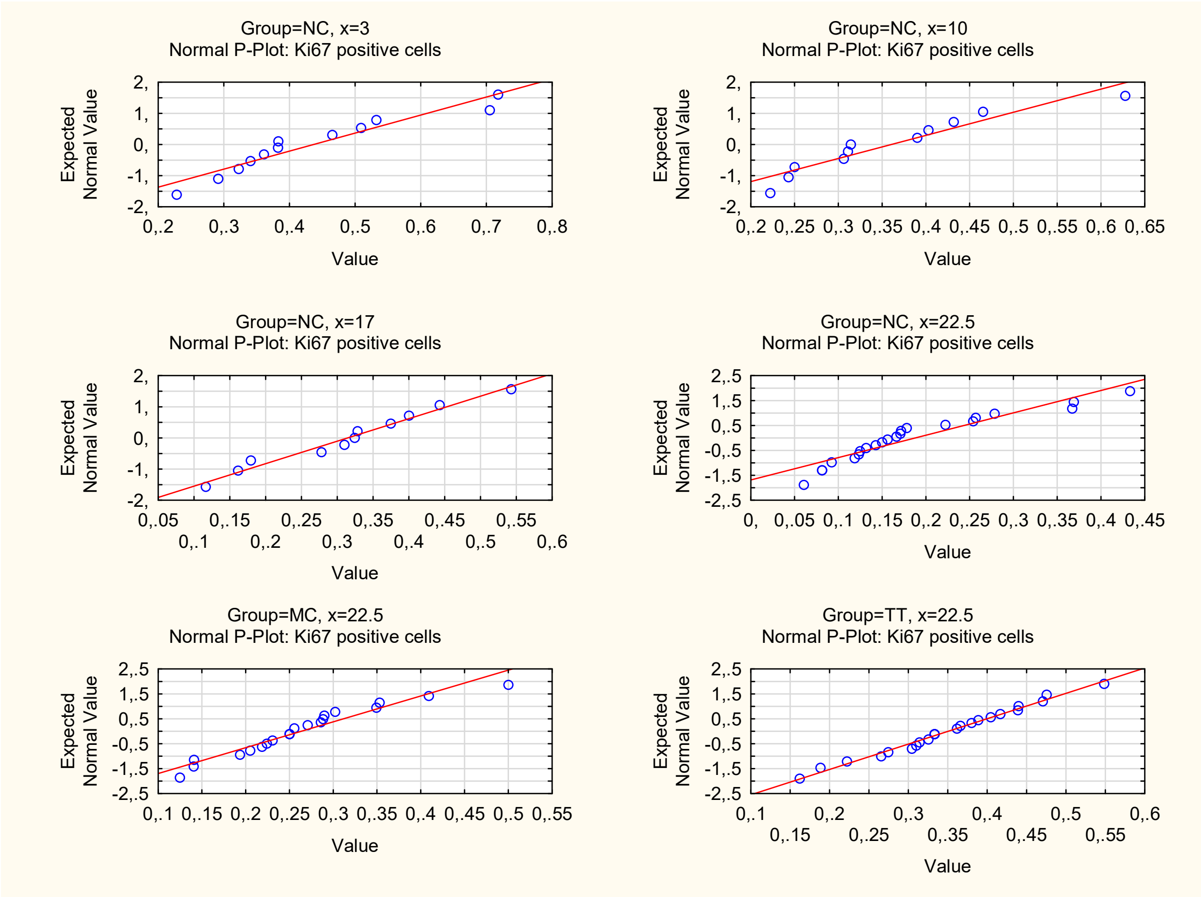

Normality test and normal probability plots suggest that we cannot reject null-hypothesis about normality of Percentage of Ki67 positive cells in all subsamples except for group NC with $x = 22.5$. Moreover, estimates of their standard deviations suggest homogeneity of variances. By Bartlett's Chi-square test [5] ($\chi^2 = 6.6299$, df = 5, pv = 0.2497), and Levene's test for homogeneity of variances [1] (F = 1.5611, dfs= (5, 94), pv = 0.1787) we cannot reject null-hypothesis that population variances of all subsamples of Y are equal. In further analysis, we will assume that

variable Percentage of Ki67 positive cells has normal distribution with equal population variances in all subsamples.

For subsamples for group NC we assume that a linear regression model

$$Y = \beta_0 + \beta_1 x + \varepsilon$$

holds with Y (Percentage of Ki67 positive cells) as dependent variable and x (Age in months) as independent variable (regressor). In addition, we can assume that random errors ε belonging to different observations are normally distributed, independent, and have equal variances (σ^2). We fitted this model to the data, and the model seemed to be significant (Test for significant regression: F = 34.4754, dfs = (1, 54), pv < 10^{-6}, see [2], §12, pp. 420-422). Moreover, since we have repeated observations of Y for each different value of the regressor, we performed lack-of-fit test ([2], §11.4, pp. 361-364). The result is that we cannot reject null-hypothesis that mean values of Y linearly depend on x (F = 0.8763, dfs = (2, 52), pv = 0.4224). Table S2 is the ANOVA table. Estimates of the model parameters (coefficients) are in Table S3.

Table S2: ANOVA table for linear regression model fitting of %Ki67 positive cells, group NC

	Analysis of Variance; DV: Ki67 positive cells Group = NC				
Effect	Sums of Squares	df	Mean Squares	F	p-value
Regress.	0.516736	1	0.516736	34.47541	0.000000
Residual	0.809381	54	0.014989		
Total	1.326118				

Table S3: Estimates of the parameters of linear regression model for %Ki67 positive cells, group NC

Parameter	Estimate	Std. error	t-value	pv
β_0	0.4855	0.0356	13.6438	$< 10^{-7}$
β_1	-0.0125	0.0021	-5.8716	$3 \cdot 10^{-7}$

Normal probability plot of residuals can be found in Figure S2, and the estimated linear model is presented in Figure 2 in the main text.

At $x = 22.5$ we compared subsamples belonging to the different groups by an ANOVA method. Table S4 is the ANOVA table.

Table S4: ANOVA table of % Ki67 positive cells for groups NC, MC, and TT at $x = 22.5$.

Effect	Univariate Results for % Ki67 positive cells; Groups: NC, MC, TT; $x = 22.5$				
	Degr. of Freedom	SS	MS	F	p-value
Group	2	0.297776	0.148888	16.6060	0.000002
Error	63	0.564852	0.008966		
Total	65	0.862628			

We can conclude that group population means differ significantly. By applying Tuckey's HSD test ([4], Test 21c, pp. 534-535) and Fisher's LSD test ([4], Test 21a, pp. 528-531) we can say which pairs of means differ significantly. The test results are presented in Table S5: all pairs of means among groups NC, MC and TT, differ significantly (at level 5%). Group means together with their standard error bars are presented in Figure 2 in the main text. Normal probability plots of the residuals are presented in Figure S2.

Table S5: Comparison of group population means of % Ki67 positive cells for groups NC, MC, and TT at $x = 22.5$, by Tuckey's HSD and Fisher's LSD tests.

Cell No.	Tukey HSD test; variable % Ki67 positive cells<br>Approximate Probabilities for Post Hoc Tests<br>Error: Between MS = .00897, df = 63.000<br>$x = 22.5$			
	Group	{1}<br>.18785	{2}<br>.26346	{3}<br>.35037
1	NC		0.029546	0.000116
2	MC	0.029546		0.009592
3	TT	0.000116	0.009592	

Cell No.	LSD test; variable % Ki67 positive cells<br>Probabilities for Post Hoc Tests<br>Error: Between MS = .00897, df = 63.000<br>$x = 22.5$			
	Group	{1}<br>.18785	{2}<br>.26346	{3}<br>.35037

1	NC		0.011087	0.000000
2	MC	0.011087		0.003433
3	TT	0.000000	0.003433	

Figure S2: Normal probability plots of % Ki67 positive cells residuals

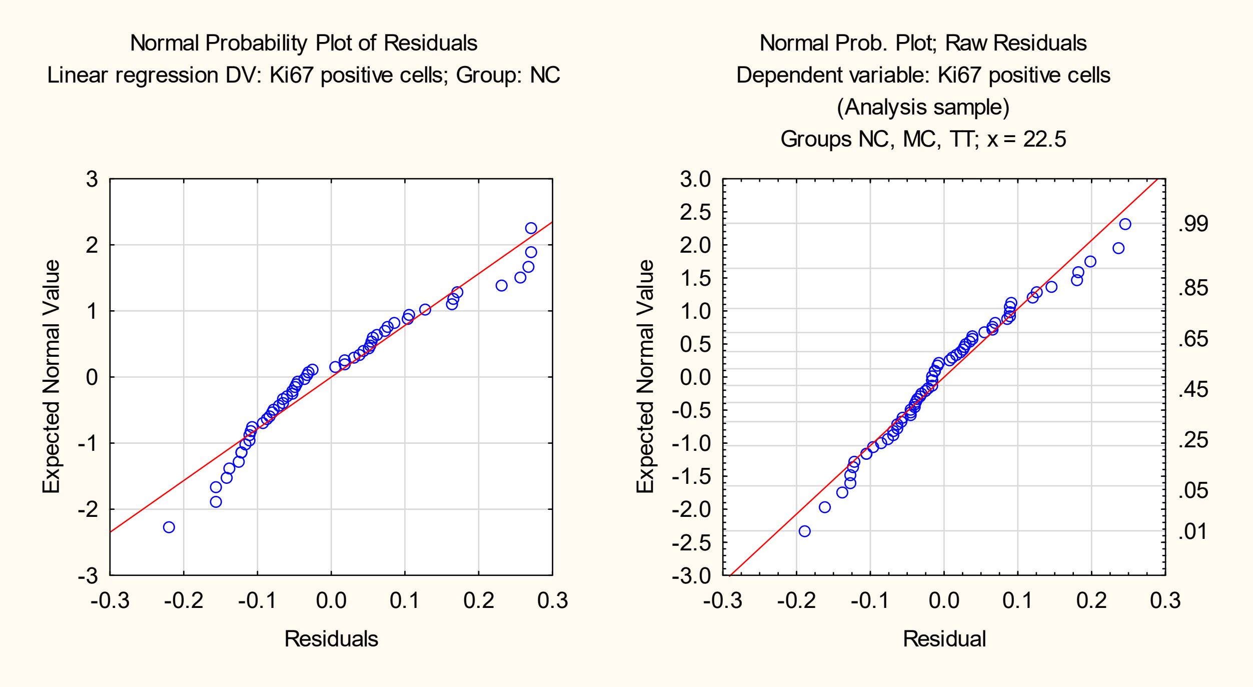

Cell density in skin

Cell density in skin was measured by calculating number of cells (Y) in a sample of skin field with dimensions 100 μm x 100 μm. The following are the same as in case of Percentage of Ki67 positive cells data. Numbers of cells were measured independently with respect to variable Age in months (x). The data were classified in three groups: untreated tissue (NC), mechanically treated tissue (MC) and with young cells treated tissue (TT). In NC class Y was measured several times (n) with respect to Age of x = 3 months, 10 months, 17 months and between 21 and 24 months (x = 22.5); in MC and TT classes Y was measured several times only for Age of x = 22.5 months. For all data subsamples, we tested normality by Lilliefors variant of Kolmogorov-Smirnov test, and by

Shapiro-Wilk test. Descriptive statistics of Y together with the normality test results for the subsamples are presented in Table S6. Normal probability plots of the subsamples are in Figure S3.

Table S6: Descriptive statistics and tests of normality for Number of cells

Group	Age in months (x)	No.of cases (n)	Mean ($\bar{Y}$)	Std. dev. (σ)	Std. error of mean	Lillie test L	pv	Shapiro -Wilk W	test pv
NC	3	9	24.694	2.666	0.889	0.2322	< 0.20	0.9337	0.5173
NC	10	8	23.481	2.585	0.914	0.2345	< 0.20	0.9230	0.4544
NC	17	9	19.343	4.084	1.361	0.2001	> 0.20	0.8943	0.2207
NC	22.5	9	18.778	4.280	1.427	0.1504	> 0.20	0.9583	0.7803
MC	22.5	9	25.806	3.682	1.227	0.1827	> 0.20	0.9423	0.6059
TT	22.5	9	35.750	10.033	3.344	0.1468	> 0.20	0.9100	0.3157

Figure S3: Normal probability plots of Number of cells subsamples

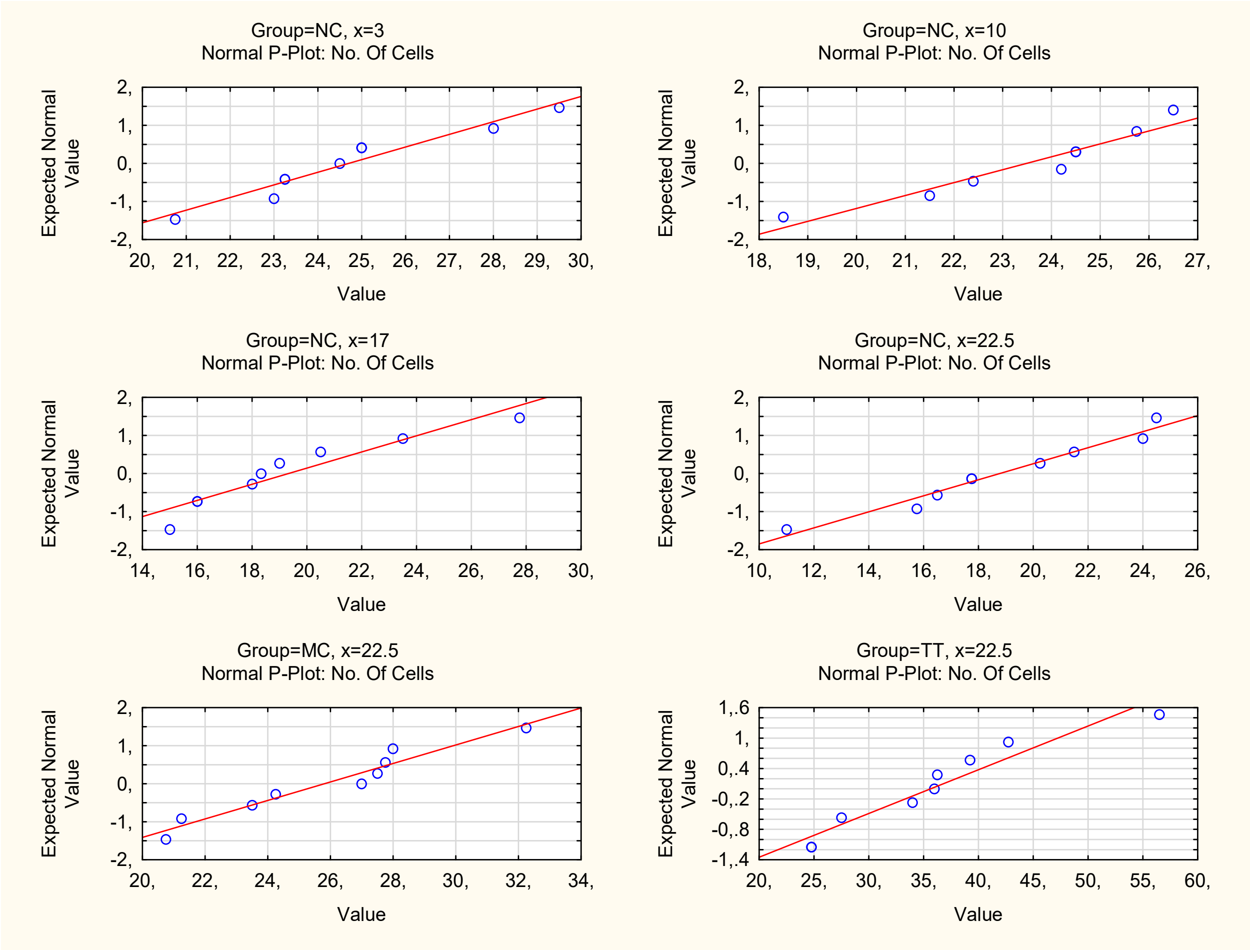

Normality test and normal probability plots suggest that we cannot reject null-hypothesis about normality of Number of cells. Moreover, estimates of their standard deviations do not suggest homogeneity of variances in all subsamples, but for subsamples for group NC they do. By Bartlett's Chi-square test ($\chi^2 = 3.0607$, df = 3, pv = 0.3824), and Levene's test for homogeneity of variances (F = 1.0802, dfs= (3, 31), pv = 0.3719) we cannot reject null-hypothesis that population variances of subsamples of Y in group NC are equal. The same conclusion about homogeneity of variances in case of subsamples with Age value $x = 22.5$ (groups NC, MC, and TT) is not so clear. Namely, by Bartlett's Chi-square test we reject null-hypothesis that their population variances are equal ($\chi^2 = 9.3825$, df = 2, pv = 0.0092), but by Levene's test for homogeneity of variances (F = 2.6515, dfs= (2, 24), pv = 0.0911) we cannot reject it at 5% level. Nevertheless, in further analysis, we will assume that variable Number of cells has normal distribution with equal population variances in subsamples of group NC with different Age values, and the same variable, but in groups NC, MC, and TT together (with the same Age value $x = 22.5$ months), has normal distribution with equal population variances too.

As same as in the former case, for subsamples for group NC we assume too that a linear regression model

$$Y = \beta_0 + \beta_1 x + \varepsilon$$

holds with Y (Number of cells) as dependent variable and x (Age in months) as independent variable (regressor). In addition, we can assume too that random errors ε belonging to different observations are normally distributed, independent, and have equal variances (σ^2). We fitted this model to the data, and the model seemed to be significant (F = 17.8893, dfs = (1, 33), pv = $1.7 \cdot 10^{-4}$). By lack-of-fit test (F = 0.6252, dfs = (2, 31), pv = 0.5418) we cannot reject null-hypothesis that

mean values of Y linearly depend on x. Table S7 is the ANOVA table. Estimates of the model parameters (coefficients) are in Table S8.

Table S7: ANOVA table for linear regression model fitting of Number of cells, group NC

Effect	Analysis of Variance; DV: No. Of Cells Group NC				
	Sums of Squares	df	Mean Squares	F	p-value
Regress.	216.3433	1	216.3433	17.88931	0.000175
Residual	399.0836	33	12.0934		
Total	615.4269				

Table S8: Estimates of the parameters of linear regression model for Number of cells, group NC

Parameter	Estimate	Std. error	t-value	pv
β_0	25.9476	1.2007	21.6111	$< 10^{-7}$
β_1	-0.3351	0.0792	-4.2296	$1.7 \cdot 10^{-4}$

Normal probability plot of residuals can be found in Figure S4, and the estimated linear model is presented in Figure 1 in the main text.

At $x = 22.5$ we compared subsamples belonging to the different groups by the ANOVA method. Table S9 is the ANOVA table.

Table S9: ANOVA table of Number of cells for groups NC, MC, and TT at $x = 22.5$.

Effect	Univariate Results for No. Of Cells; Groups: NC, MC, TT; x = 22.5				
	Degr. of Freedom	SS	MS	F	p-value
Group	2	1309.01	654.51	14.8151	0.000065
Error	24	1060.28	44.18		
Total	26	2369.29			

It turns out that group population means differ significantly. We applied Tuckey's HSD test and Fisher's LSD test to determine which pairs of means differ significantly. The test results are presented in Table S10: all pairs of means among groups NC, MC and TT, differ significantly at

level 5% except one pair by Tuckey's HSD test which differ significantly at level 10%. Group means together with their standard error bars are presented in Figure 1 in the main text. Normal probability plots of the residuals are presented in Figure S4.

Table S10: Comparison of group population means of Number of cells for groups NC, MC, and TT at $x = 22.5$, by Tuckey's HSD and Fisher's LSD tests.

Cell No.	Tukey HSD test; variable No. Of Cells<br>Approximate Probabilities for Post Hoc Tests<br>Error: Between MS = 44.178, df = 24.000<br>x = 22.5			
	Group	{1}<br>18.778	{2}<br>25.806	{3}<br>35.750
1	NC		0.084222	0.000168
2	MC	0.084222		0.011096
3	TT	0.000168	0.011096	
Cell No.	LSD test; variable No. Of Cells<br>Probabilities for Post Hoc Tests<br>Error: Between MS = 44.178, df = 24.000<br>x = 22.5			
	Group	{1}<br>18.778	{2}<br>25.806	{3}<br>35.750
1	NC		0.034401	0.000015
2	MC	0.034401		0.004092
3	TT	0.000015	0.004092	

Figure S4: Normal probability plots of Number of cells residuals

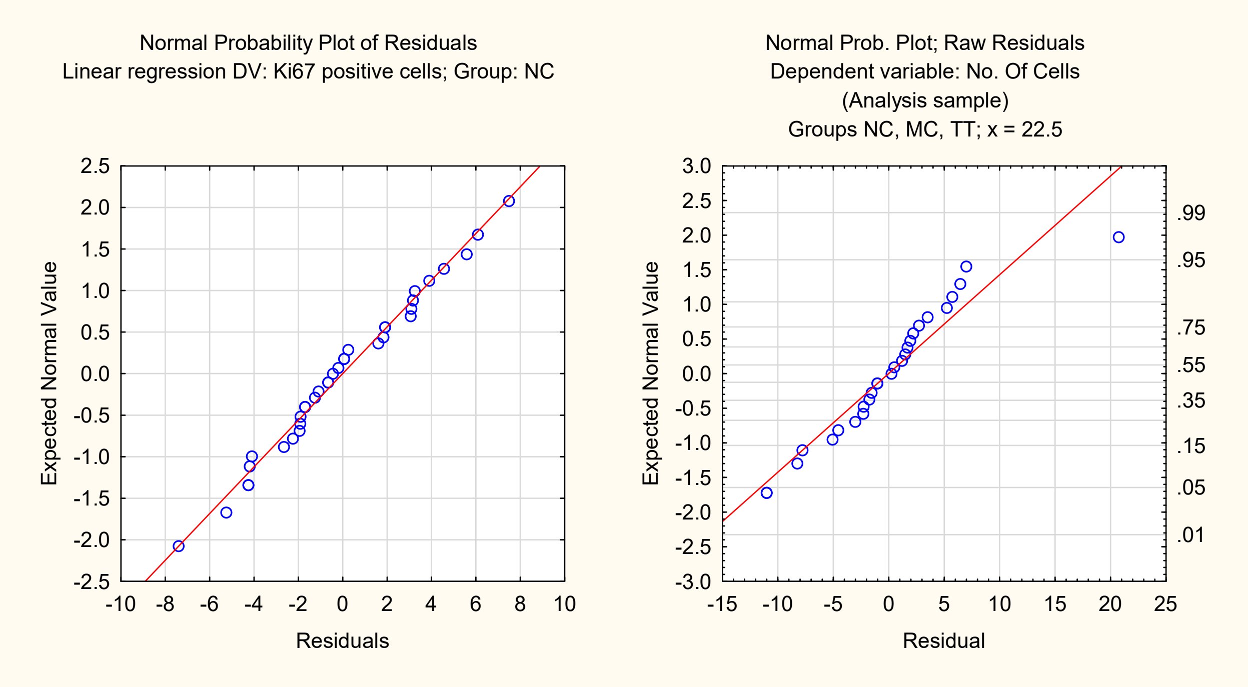

Average cell distance in skin

Average cell distance (briefly, Distance in plots) is a derivative of Cell density in skin obtained by the following formula:

$$\text{Average cell distance} = \frac{100\mu m}{\sqrt{\text{Number of cells}}} \cdot \frac{1+\sqrt{2}}{2}.$$

We have obtained this formula by the following rationality. We assume that the observational square window (with dimensions $100\mu m$ x $100\mu m$) contains Number of cells small average identical squares with sides equal to a such that each small square contains a single cell in its center. By equalizing the total area of the window and the total area of the small squares in the window:

$$(100\mu m)^2 = (\text{Number of cells})\cdot a^2,$$

we obtain that side a of a small average square is equal to:

$$a = \frac{100\mu m}{\sqrt{\text{Number of cells}}}.$$

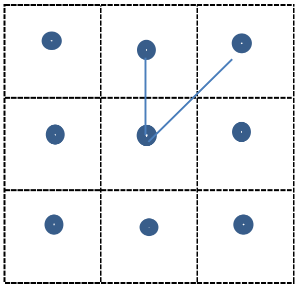

By a symmetry argument and an assumption that Number of cells is an enough big number, we can conclude that average distance $\bar{d}$ between two cells is approximately equal to the average of distances from the (two) adjacent cells (see picture above):

$$\bar{d} = \frac{a+a\sqrt{2}}{2} = a \cdot \frac{1+\sqrt{2}}{2} = \frac{100\mu m}{\sqrt{\text{Number of cells}}} \cdot \frac{1+\sqrt{2}}{2}.$$

Hence the formula for Average cell distance = $\bar{d}$, follows.

Since Number of cells were measured independently with respect to variable Age in months (x) and the data were classified in three groups: untreated tissue (NC), mechanically treated tissue (MC) and with young cells treated tissue (TT), we classified Average cell distance with respect to the same groups and the same Age in months values as in former variable cases. Here, the dependent variable Y is Average cell distance. Descriptive statistics of Y together with the normality test results for the subsamples are presented in Table S11. Normal probability plots of the subsamples are in Figure S5.

Table S11: Descriptive statistics and tests of normality for Average cell distance

Group	Age in months (x)	No.of cases (n)	Mean ($\bar{Y}$)	Std. dev. (σ)	Std. error of mean	Lillie test L	pv	Shapiro-Wilk test W	pv
NC	3	9	24.412	1.286	0.429	0.2034	> 0.20	0.9516	0.7074
NC	10	8	25.049	1.492	0.528	0.2516	< 0.15	0.8838	0.2045
NC	17	9	27.848	2.668	0.889	0.1484	> 0.20	0.9480	0.6685
NC	22.5	9	28.447	3.682	1.227	0.1818	> 0.20	0.8961	0.2303

MC	22.5	9	23.954	1.724	0.575	0.2123	> 0.20	0.9377	0.5574
TT	22.5	9	20.714	2.745	0.915	0.1649	> 0.20	0.9395	0.5769

Figure S5: Normal probability plots of Average cell distance subsamples

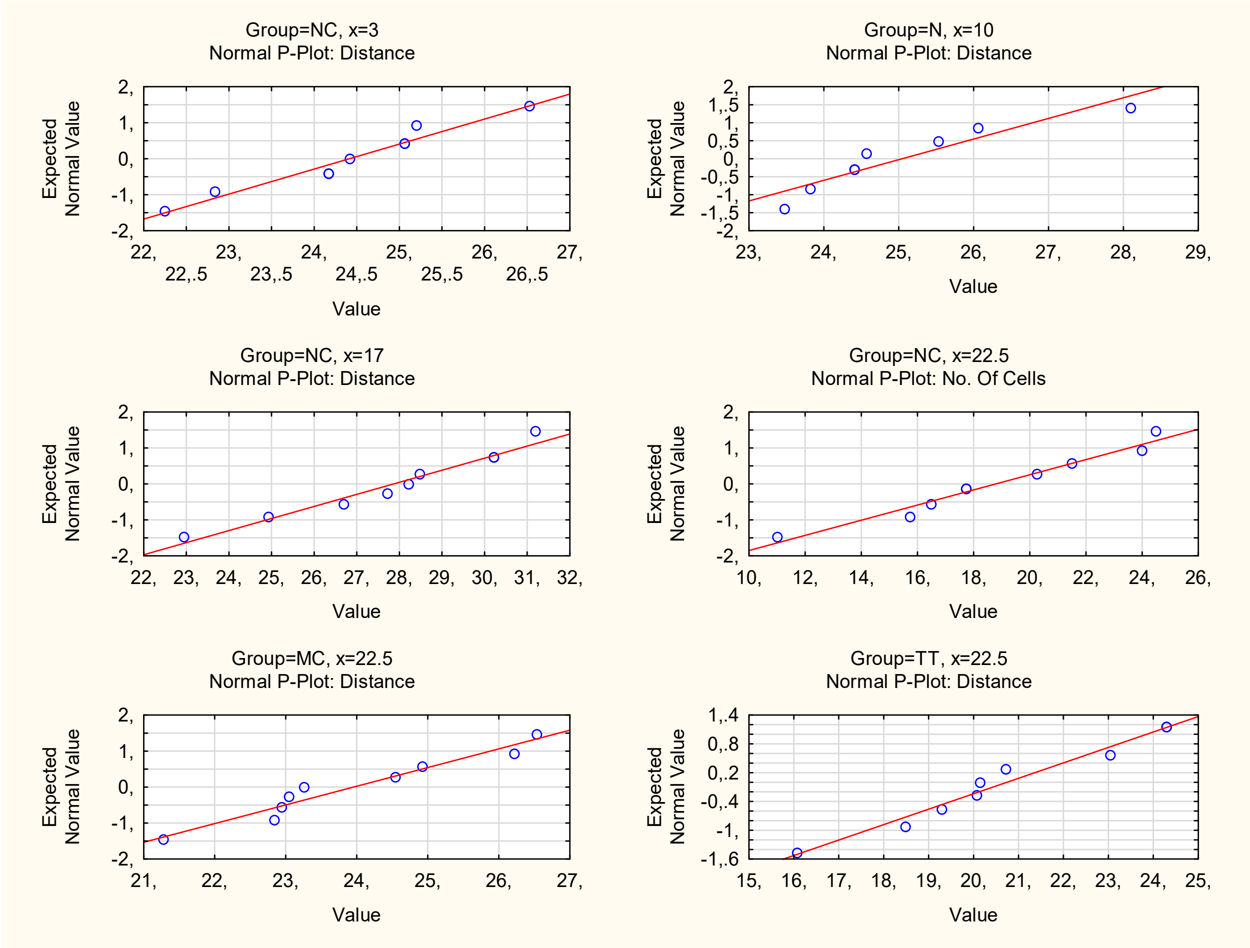

Normality tests and normal probability plots suggest that we cannot reject null-hypothesis about normality of Average cell distance. By Levene's test for homogeneity of variances (F = 1.6851, dfs= (5, 47), pv = 0.1567) we cannot reject null-hypothesis that population variances of all subsamples of Y in group NC are equal although Bartlett's Chi-square test (χ^2 = 11.9400, df = 5, pv = 0.0356) suggest that we can reject this hypothesis at 5% level, but not at 1% level of

significance. Nevertheless, in further analysis we will assume that variable Average cell distance has normal distribution with equal population variances in all subsamples.

As same as in the former case, for subsamples for group NC we assume too that a linear regression model

$$Y = \beta_0 + \beta_1 x + \varepsilon$$

holds with Y (Average cells distance) as dependent variable and x (Age in months) as independent variable (regressor). In addition, we can assume too that random errors ε belonging to different observations are normally distributed, independent, and have equal variances (σ^2). We fitted this model to the data, and the model seemed to be significant (F = 16.3246, dfs = (1, 33), pv = $3.0 \cdot 10^{-4}$). By lack-of-fit test (F = 0.5595, dfs = (2, 31), pv = 0.5772) we cannot reject null-hypothesis that mean values of Y linearly depend on x. Table S12 is the ANOVA table. Estimates of the model parameters (coefficients) are in Table S13.

Table S12: ANOVA table for linear regression model fitting of Average cell distance, group NC

Effect	Analysis of Variance; DV: Distance Group NC				
	Sums of Squares	df	Mean Squares	F	p-value
Regress.	99.5303	1	99.53029	16.32461	0.000300
Residual	201.1993	33	6.09695		
Total	300.7296				

Table S13: Estimates of the parameters of linear regression model for Average cell distance, group NC

Parameter	Estimate	Std. error	t-value	pv
β_0	23.4754	0.8525	27.5366	$< 10^{-7}$
β_1	0.2273	0.0563	4.0404	$3.0 \cdot 10^{-4}$

Normal probability plot of residuals can be found in Figure S6, and the estimated linear model is presented in Figure 1 in the main text.

At *x* = 22.5 we compared subsamples belonging to the different groups by the ANOVA method. Table S14 is the ANOVA table.

Table S14: ANOVA table of Average cell distance for groups NC, MC, and TT at *x* = 22.5.

Effect	Univariate Results for Average cell distance; Groups: NC, MC, TT; x = 22.5				
	Degr. of Freedom	SS	MS	F	p-value
Group	2	271.4435	135.7218	16.9212	0.000026
Error	24	192.4990	8.0208		
Total	26	463.9426			

It turns out that group population means differ significantly. The results of Tuckey's HSD and Fisher's LSD tests are presented in Table S15: all pairs of means among groups NC, MC and TT, differ significantly at level 5% except one pair by Tuckey's HSD test which differ significantly at level 10%. Group means together with their error bars are presented in Figure 1 in the main text. Normal probability plots of the residuals are presented in Figure S6.

Table S15: Comparison of group population means of Average cell distance for groups NC, MC, and TT at *x* = 22.5, by Tuckey's HSD and Fisher's LSD tests.

Cell No.	Tukey HSD test; variable Distance<br>Approximate Probabilities for Post Hoc Tests<br>Error: Between MS = 8.0208, df = 24.000<br>x = 22.5			
	Group	{1}<br>28.447	{2}<br>23.954	{3}<br>20.714
1	NC		0.007078	0.000141
2	MC	0.007078		0.058016
3	TT	0.000141	0.058016	
Cell No.	LSD test; variable Distance P<br>robabilities for Post Hoc Tests<br>Error: Between MS = 8.0208, df = 24.000<br>x = 22.5			

	Group	{1}<br>28.447	{2}<br>23.954	{3}<br>20.714
1	NC		0.002568	0.000006
2	MC	0.002568		0.023099
3	TT	0.000006	0.023099	

Figure S6: Normal probability plots of Average cell distance residuals

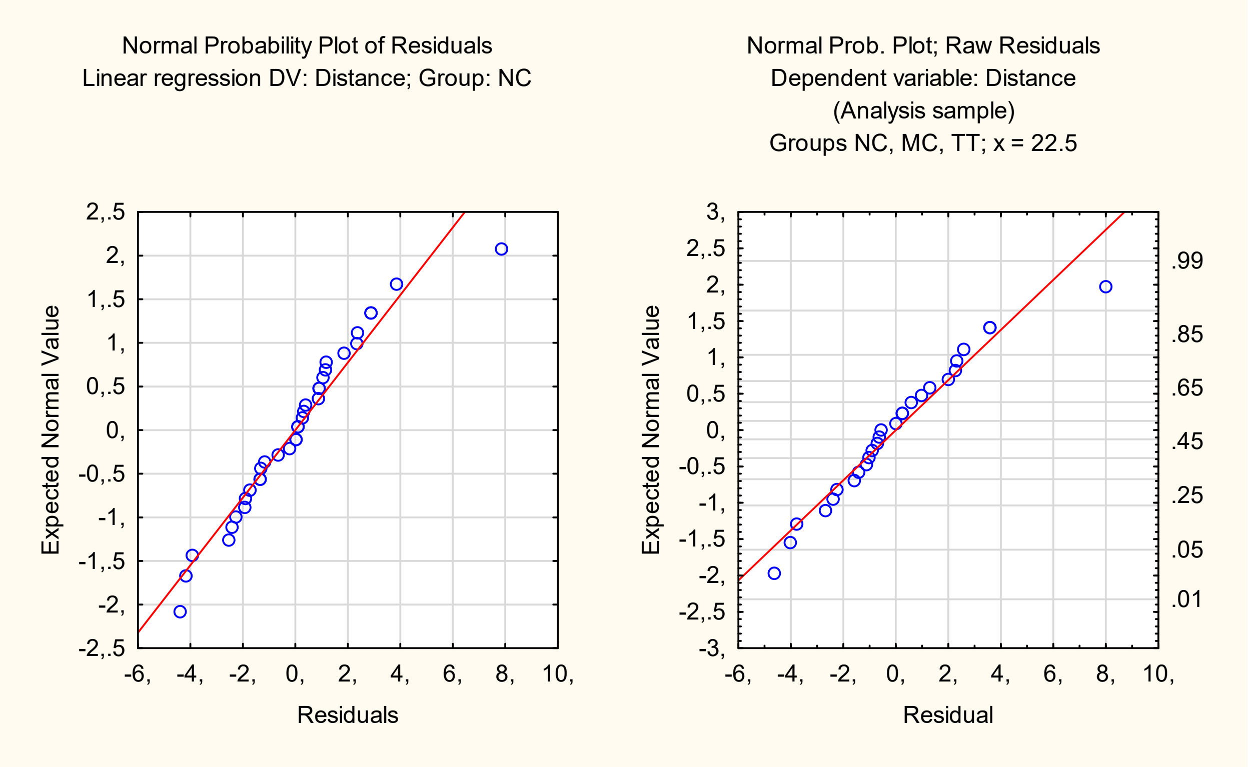

Immunofluorescence signals of collagen I

Signals of collagen I (briefly, Coll. I in plots and tables) were measured several times independently with respect to the same values of variable Age in months (x), and the data were classified in the same three groups: untreated tissue (NC), mechanically treated tissue (MC) and with young cells treated tissue (TT) as in cases of all former analyzed variables. Here, the dependent variable Y is Signals of collagen I. Descriptive statistics of log-transformed variable Y

(log$Y$) together with the normality test results for the subsamples are presented in Table S21. Normal probability plots of the subsamples are in Figure S9.

Table S16: Descriptive statistics and tests of normality for Log Signals of collagen I

Group	Age in months (x)	No.of cases (n)	Mean ($\overline{\log Y}$)	Std. dev. (σ)	Std. error of mean	Lillie test L	pv	Shapiro -Wilk test W	pv
NC	3	20	11.647	0.204	0.046	0.1684	< 0.15	0.9318	0.1671
NC	10	27	11.440	0.370	0.071	0.0810	> 0.20	0.9783	0.8223
NC	17	24	11.563	0.344	0.070	0.1482	> 0.20	0.9452	0.2129
NC	22.5	27	11.978	0.456	0.088	0.1672	< 0.05	0.9305	0.0711
MC	22.5	27	11.561	0.383	0.074	0.2238	< 0.01	0.8981	0.0121
TT	22.5	27	11.633	0.307	0.059	0.1461	< 0.10	0.9506	0.2210

Figure S7: Mean plots with error bars of Log Signals of collagen I subsamples

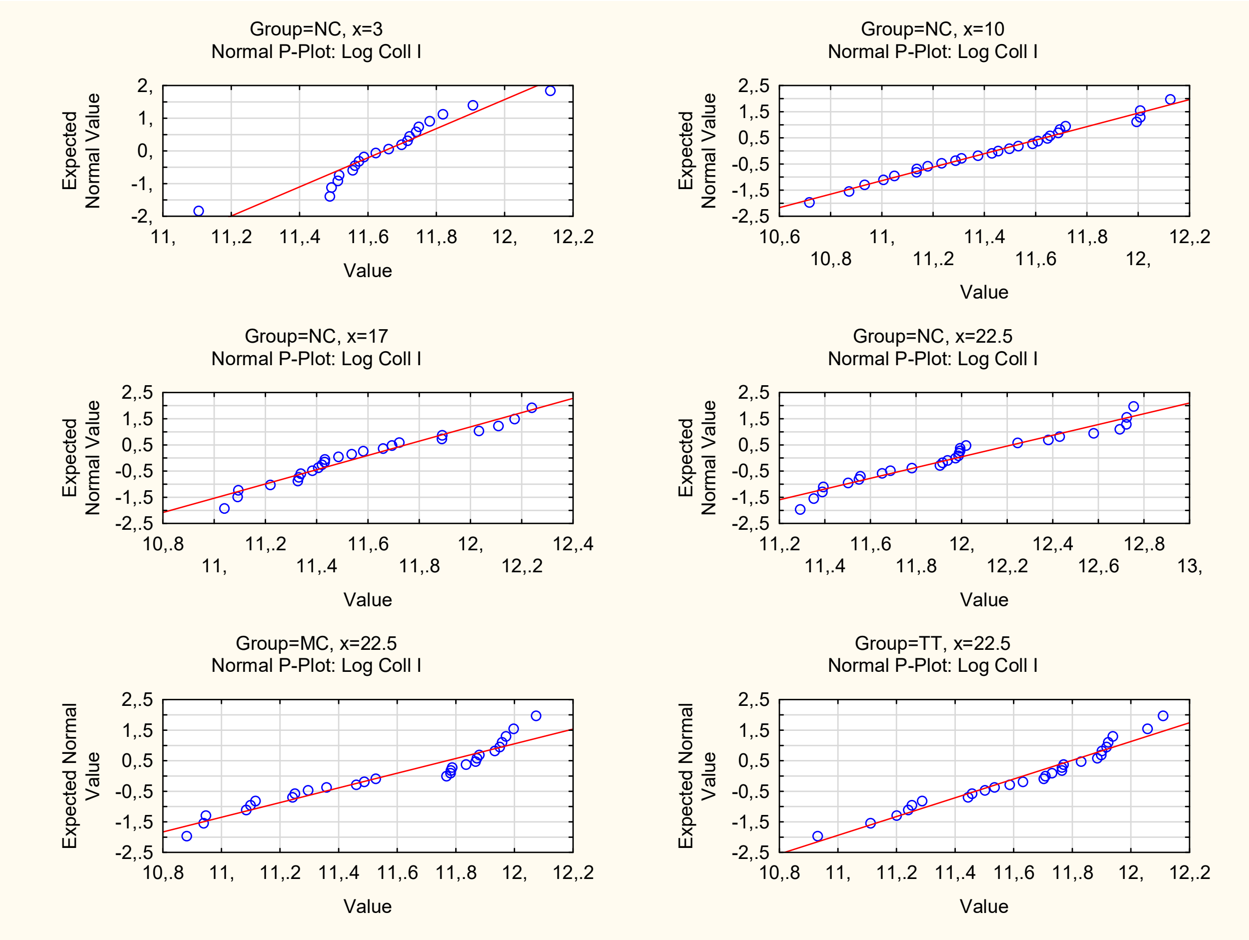

Normality tests and normal probability plots suggest that we cannot reject null-hypothesis about normality of Log Signals of collagen I in almost all subsamples except probably in cases $x = 3$ and $x = 22.5$ of Group NC, and in case $x = 22.5$ of Group MC. By Bartlett's Chi-square test (χ^2 = 13.4807, df = 5, pv = 0.0193) and Levene's test for homogeneity of variances (F = 3.2823, dfs= (5, 146), pv = 0.0077) we reject null-hypothesis that population variances of all subsamples of log$Y$ are equal at 5% level. Hence, in further analysis we will assume that transformed variable Log Signals of collagen I has normal distribution with unequal population variances among subsamples.

For subsamples for group NC and log-transformed variable Log Signals of collagen I (log$Y$) we assume that a quadratic regression model

$$\log Y = \beta_0 + \beta_1 x + \beta_2 x^2 + \varepsilon$$

holds with Age in months as independent variable x (regressor). In addition, we can assume too that random errors ε belonging to different observations are normally distributed, independent, and have generally different variances (σ_x^2) for different x values. We fitted this model to the data by a weighted least-square method with weights equal to $w = 1/\sigma_x^2$ (variances σ_x^2 were estimated from the subsamples). The model was significant (F = 11.9823, dfs = (2, 95), pv = $2\cdot10^{-5}$). By lack-of-fit test, modified for the used weights ($\chi2$ = 0.1138, df = 1, pv ≈ 0.7359) we cannot reject null-hypothesis that mean values of log$Y$ quadraticly depend on x. Moreover, we cannot reduce the quadratic model to a linear model (F = 20.7652, dfs = (1, 95), pv = $2\cdot10^{-5}$). Table S22 contains ANOVA tables for the quadratic regression model fit. Estimates of the model parameters are in Table S23.

Table S17: ANOVA tables for quadratic regression model fitting of Log Signals of collagen I, group NC

Effect	Univariate Results Log Coll I<br>Regression<br>Sigma-restricted parameterization Effective hypothesis decomposition<br>Include cases: 1:98<br>Weight variable: w				
	Degr. of Freedom	Log Coll I SS	Log Coll I MS	Log Coll I F	Log Coll I p
Intercept	1	19555.42	19555.42	19739.56	0.000000
Age in months	1	16.43	16.43	16.59	0.000096
Age in months^2	1	20.57	20.57	20.77	0.000015
Error	95	94.11	0.99		
Total	97	117.85			

Table S18: Estimates of the parameters of quadratic regression model for Log Signals of collagen I, group NC

Parameter	Estimate	Std. error	t-value	pv
β_0	11.8611	0.0844	140.4975	0
β_1	-0.0823	0.0202	-4.0728	0.00010
β_2	0.0039	0.0008	4.5569	0.00002

Normal probability plot of residuals can be found in Figure S10, and the estimated quadratic model is presented in Figure 3 in the main text.

At $x = 22.5$ we compared subsamples of Log Signals of collagen I belonging to the different groups by the ANOVA method. By Bartlett's Chi-square test ($\chi 2 = 3.9312$, df = 2, pv = 0.1401) and Levene's test for homogeneity of variances (F = 1.9274, dfs= (2, 78), pv = 0.1524) we cannot reject null-hypothesis that population variances of the subsamples of log$Y$ are equal. Hence, we can assume that log$Y$ has normal laws with equal population variances for each groups at $x = 22.5$. Table S24 is the ANOVA table.

Table S19: ANOVA table of Log Signals of collagen I for groups NC, MC, and TT at $x = 22.5$.

	Univariate Results for Log Signals of collagen I; Groups: NC, MC, TT; x = 22.5				
Effect	Degr. of Freedom	SS	MS	F	p-value
Group	2	2.6821	1.3410	8.9557	0.0003
Error	78	11.6799	0.1497		
Total	80	14.3620			

It turns out that group population means differ significantly. The results of Tuckey's HSD and Fisher's LSD tests are presented in Table S25: group NC mean of log$Y$ differs significantly from means of groups MC and TT, but groups MC and TT means do not differ significantly from each other. Group means of log$Y$ together with their error bars are presented in Figure 3 in the main text. Normal probability plots of the residuals are presented in Figure S10.

Table S20: Comparison of group population means of Log Signals of collagen I for groups NC, MC, and TT at $x = 22.5$, by Tuckey's HSD and Fisher's LSD tests.

	Tukey HSD test; variable Log Signals of collagen I<br>Approximate Probabilities for Post Hoc Tests<br>Error: Between MS = .14974, df = 78.000<br>x = 22.5			
Cell No.	Group	{1}<br>11.978	{2}<br>11.561	{3}<br>11.633
1	NC		0.000574	0.004511
2	MC	0.000574		0.776086
3	TT	0.004511	0.776086	
	LSD test; variable Log Signals of collagen I<br>Probabilities for Post Hoc Tests<br>Error: Between MS = .14974, df = 78.000<br>x = 22.5			
Cell No.	Group	{1}<br>11.978	{2}<br>11.561	{3}<br>11.633
1	NC		0.000166	0.001564
2	MC	0.000166		0.498730
3	TT	0.001564	0.4987330	

Figure S8: Normal probability plots of Log Signals of collagen I residuals

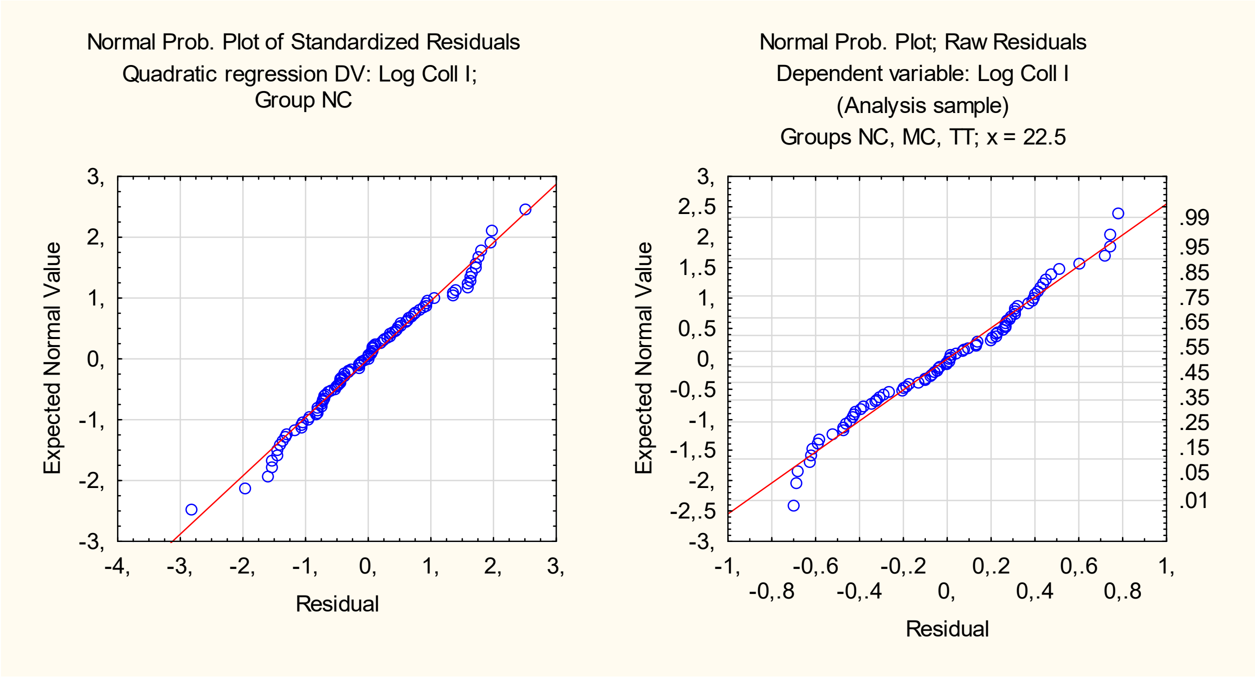

Immunofluorescence signals of collagen III

Signals of collagen III (briefly, Coll. III in plots and tables) were measured several times independently with respect to the same values of variable Age in months (x), and the data were classified in the same three groups: untreated tissue (NC), mechanically treated tissue (MC) and with young cells treated tissue (TT) as in cases of all former analyzed variables. Here, the dependent variable Y is Signals of collagen III. Descriptive statistics of log-transformed variable Y (log$Y$) together with the normality test results for the subsamples are presented in Table S21. Normal probability plots of the subsamples are in Figure S9.

Table S21: Descriptive statistics and tests of normality for Log Signals of collagen III

Group	Age in months (x)	No.of cases (n)	Mean ($\overline{\log Y}$)	Std. dev. (σ)	Std. error of mean	Lillie test L	pv	Shapiro-Wilk test W	pv
NC	3	20	9.8681	0.7598	0.1699	0.1718	< 0.15	0.8419	0.0039
NC	10	27	10.8519	0.6187	0.1191	0.1464	< 0.10	0.9205	0.0406
NC	17	24	10.4702	1.2432	0.2538	0.1299	> 0.20	0.9575	0.3900
NC	22.5	27	11.7835	1.1030	0.2123	0.2844	< 0.01	0.8240	0.0004
MC	22.5	27	10.9251	0.9346	0.1799	0.1760	< 0.05	0.9009	0.0140
TT	22.5	27	10.0259	0.8861	0.1705	0.0988	> 0.20	0.9594	0.3571

Figure S9: Mean plots with error bars of Log Signals of collagen III subsamples

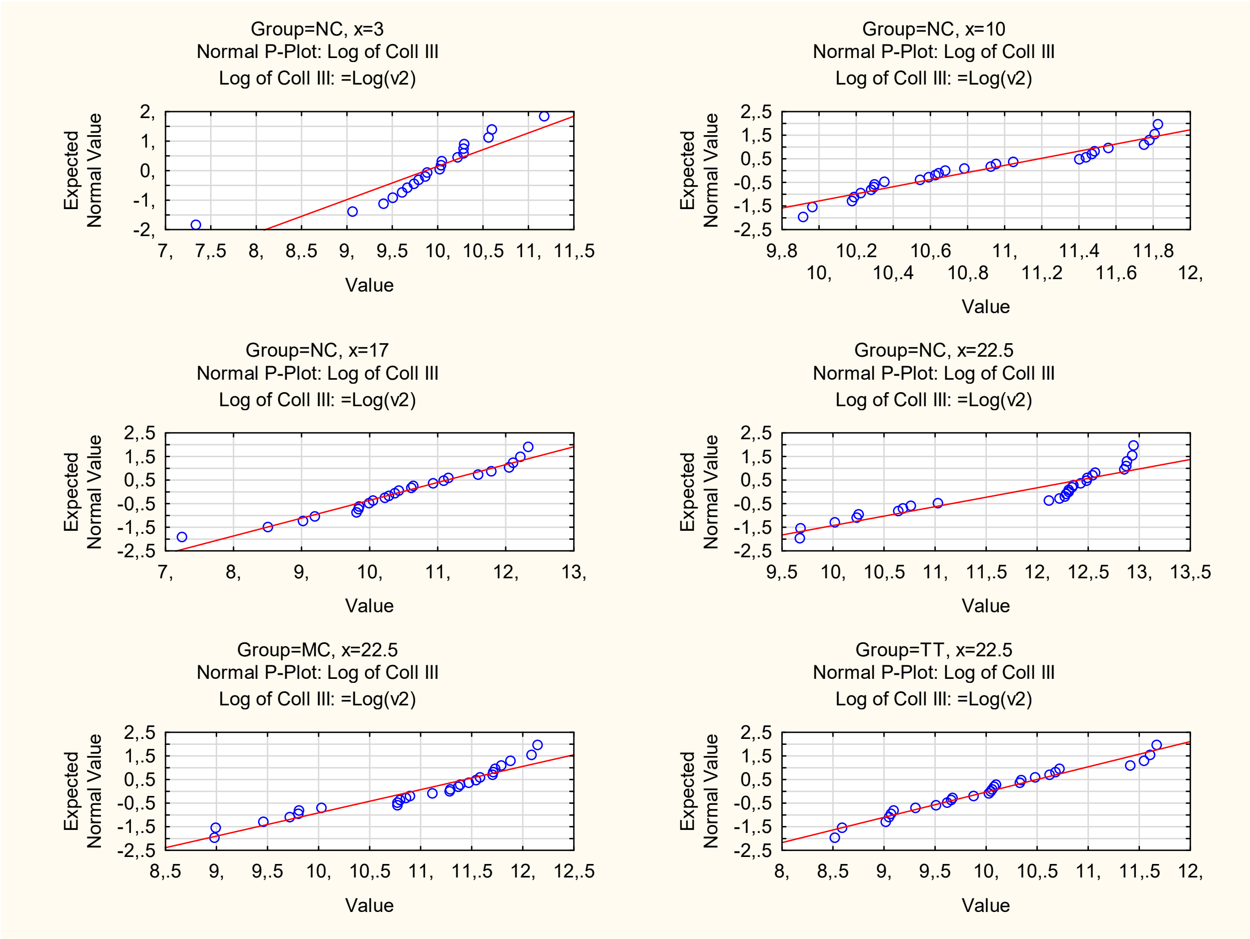

Normality tests and normal probability plots suggest that we can reject null-hypothesis about normality of Log Signals of collagen III in almost all subsamples except in cases $x = 17$ of Group NC and $x = 22.5$ of Group TT, and probably in cases $x = 10$ of Group NC and $x = 22.5$ of Group

MC (at significance level of 1%). By Bartlett's Chi-square test (χ^2 = 14.2927, df = 5, pv = 0.0139) and Levene's test for homogeneity of variances (F = 3.3005, dfs= (5, 146), pv = 0.0075) we reject null-hypothesis that population variances of all subsamples of log*Y* are equal at 5% level. Nevertheless, in further analysis we will assume that transformed variable Log Signals of collagen III has normal distribution with unequal population variances among subsamples of Group NC.

For subsamples for group NC and log-transformed variable Log Signals of collagen III (log*Y*) we assume that a linear regression model

$$\log Y = \beta_0 + \beta_1 x + \varepsilon$$

holds with Age in months as independent variable x (regressor). In addition, we can assume too that random errors ε belonging to different observations are normally distributed, independent, and have generally different variances (σ_x^2) for different x values. We fitted this model to the data by a weighted least-square method with weights equal to $w = 1/\ \sigma_x^2$ (variances σ_x^2 were estimated from the subsamples). The model was significant (F = 34.8675, dfs = (1, 96), pv = $5{\cdot}10^{-8}$). By lack-of-fit test, modified for the used weights ($\chi 2$ = 13.4220, df = 2, pv ≈ 0.0012) we reject null-hypothesis that mean values of log*Y* linearly depend on x. Nevertheless, linear regression model is still good enough for explaining a dependency of log*Y* on x, and the simplest one for this purpose. Table S22 contains ANOVA tables for the linear regression model fit. Estimates of the model parameters are in Table S23.

Table S22: ANOVA tables for quadratic regression model fitting of Log Signals of collagen III, group NC

	Analysis of Variance;<br>DV: Log of Coll III, Group = NC<br>Include cases: 1:98<br>Weight variable: w				
Effect	Sums of Squares	df	Mean Squares	F	p-value
Regress.	39.0160	1	39.01602	34.86752	$< 10^{-6}$
Residual	107.4220	96	1.11898		

Total	146.4380				

Table S23: Estimates of the parameters of quadratic regression model for Log Signals of collagen III, group NC

Parameter	Estimate	Std. error	t-value	pv
β_0	9.8126	0.1768	55.4907	$< 10^{-6}$
β_1	0.0821	0.0139	5.9049	$< 10^{-6}$

Normal probability plot of residuals can be found in Figure S10, and the estimated linear model is presented in Figure 4 in the main text.

At $x = 22.5$ we compared subsamples of Log Signals of collagen III belonging to the different groups by the ANOVA method. By Bartlett's Chi-square test ($\chi 2 = 1.3709$, df = 2, pv = 0.5039) and Levene's test for homogeneity of variances (F = 2.1130, dfs= (2, 78), pv = 0.1277) we cannot reject null-hypothesis that population variances of the subsamples of log$Y$ are equal. Hence, we can assume that log$Y$ has normal laws with equal population variances for each group at $x = 22.5$. Table S24 is the ANOVA table.

Table S24: ANOVA table of Log Signals of collagen III for groups NC, MC, and TT at $x = 22.5$.

	Univariate Results for Log Signals of collagen III; Groups: NC, MC, TT; x = 22.5				
Effect	Degr. of Freedom	SS	MS	F	p-value
Group	2	41.7125	20.8562	21.7612	$3 \cdot 10^{-8}$
Error	78	74.7564	0.9584		
Total	80	116.4689			

It turns out that group population means differ significantly. The results of Tuckey's HSD and Fisher's LSD tests are presented in Table S25: all group mean pairs differ significantly from each other. Group means of log$Y$ together with their error bars are presented in Figure 4 in the main text. Normal probability plots of the residuals are presented in Figure S10.

Table S25: Comparison of group population means of Log Signals of collagen III for groups NC, MC, and TT at $x = 22.5$, by Tuckey's HSD and Fisher's LSD tests.

	Tukey HSD test; variable Log of Coll III<br>Approximate Probabilities for Post Hoc Tests<br>Error: Between MS = .95841, df = 78.000<br>x = 22.5			
Cell No.	Group	{1}<br>11.784	{2}<br>10.925	{3}<br>10.026
1	NC		0.005326	0.000109
2	MC	0.005326		0.003364
3	TT	0.000109	0.003364	
	LSD test; variable Log of Coll III<br>Probabilities for Post Hoc Tests<br>Error: Between MS = .95841, df = 78.000<br>x = 22.5			
Cell No.	Group	{1}<br>11.784	{2}<br>10.925	{3}<br>10.026
1	NC		0.001860	0.000000
2	MC	0.001860		0.001153
3	TT	0.000000	0.001153	

Figure S10: Normal probability plots of Log Signals of collagen III residuals

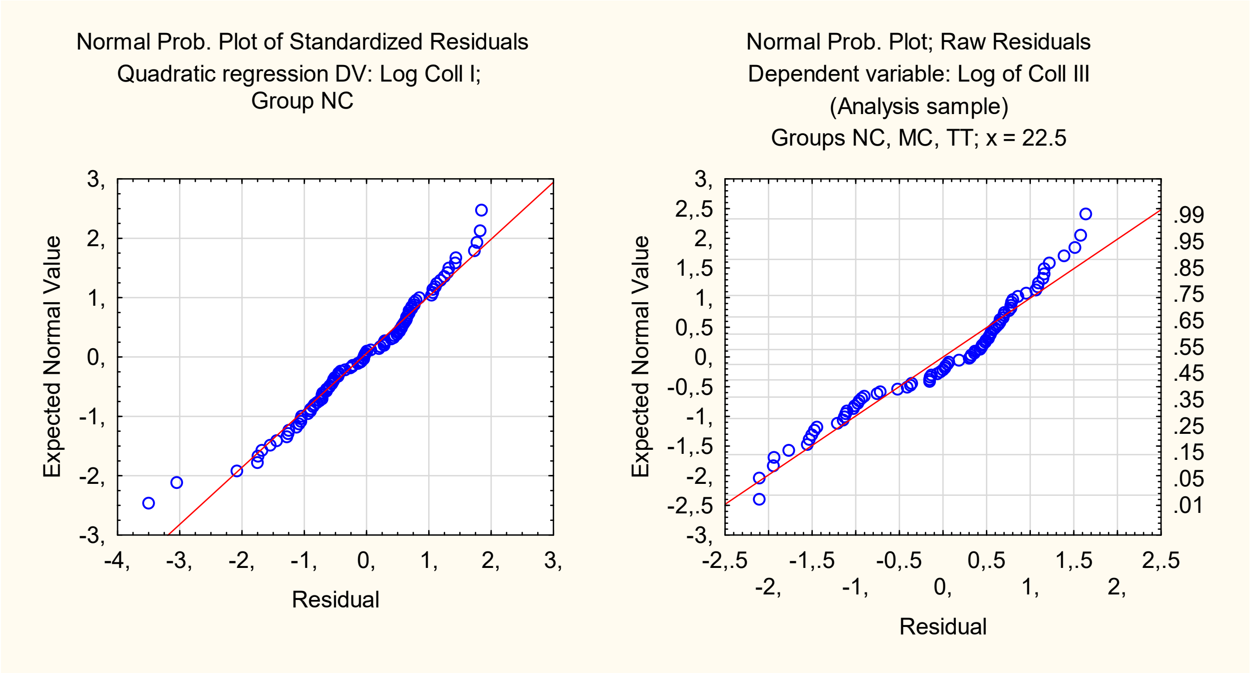

Comparison of percentage of Ki67 positive cells dynamics among groups

Percentage of Ki67 positive cells (*Y*) was measured independently among three groups: untreated tissue (negative control, NC), mechanically treated tissue (mechanical control, MC) and with young cells treated tissue (TT), with respect to variable Age in months (*x*) as a covariate (or regressor). In all groups *Y* was measured several times (*n*) at each of the following values of Age: 6 months, 12 months, 18 months and 24 months.

For all data subsamples, we tested normality by Lilliefors variant of Kolmogorov-Smirnov test, and by Shapiro-Wilk tets. Descriptive statistics of *Y* together with the normality test results for the subsamples are presented in Table S26. Normal probability plots of the subsamples are in Figure S11.

Table S26: Descriptive statistics and tests of normality for percentage of Ki67 positive cells

Group	Age in months (x)	No.of cases (n)	Mean ($\bar{Y}$)	Std. dev. (σ)	Std. error of mean	Lillie test L	pv	Shapiro-Wilk test W	pv
NC	6	6	0.2381	0.0280	0.0114	0.1727	> 0.20	0.9268	0.5560
NC	12	6	0.1884	0.0618	0.0252	0.2278	> 0.20	0.9136	0.4607
NC	18	6	0.1572	0.0762	0.0311	0.2620	> 0.20	0.8033	0.0629
NC	24	5	0.1361	0.0524	0.0234	0.3421	< 0.05	0.8326	0.1454
MC	6	6	0.2270	0.0595	0.0243	0.2220	> 0.20	0.9124	0.4521
MC	12	5	0.1925	0.0267	0.0120	0.3511	< 0.05	0.7460	0.0273
MC	18	6	0.1987	0.0642	0.0262	0.1666	> 0.20	0.9577	0.8020
MC	24	5	0.2081	0.0420	0.0188	0.2550	> 0.20	0.9194	0.5259
TT	6	6	0.2170	0.0260	0.0106	0.2631	> 0.20	0.9384	0.6459
TT	12	6	0.2320	0.0533	0.0217	0.2909	< 0.15	0.7940	0.0519
TT	18	6	0.2337	0.0767	0.0313	0.1946	> 0.20	0.9145	0.4665
TT	24	5	0.2641	0.0522	0.0234	0.1976	> 0.20	0.9296	0.5937

Figure S11: Normal probability plots of % Ki67 positive cells subsamples

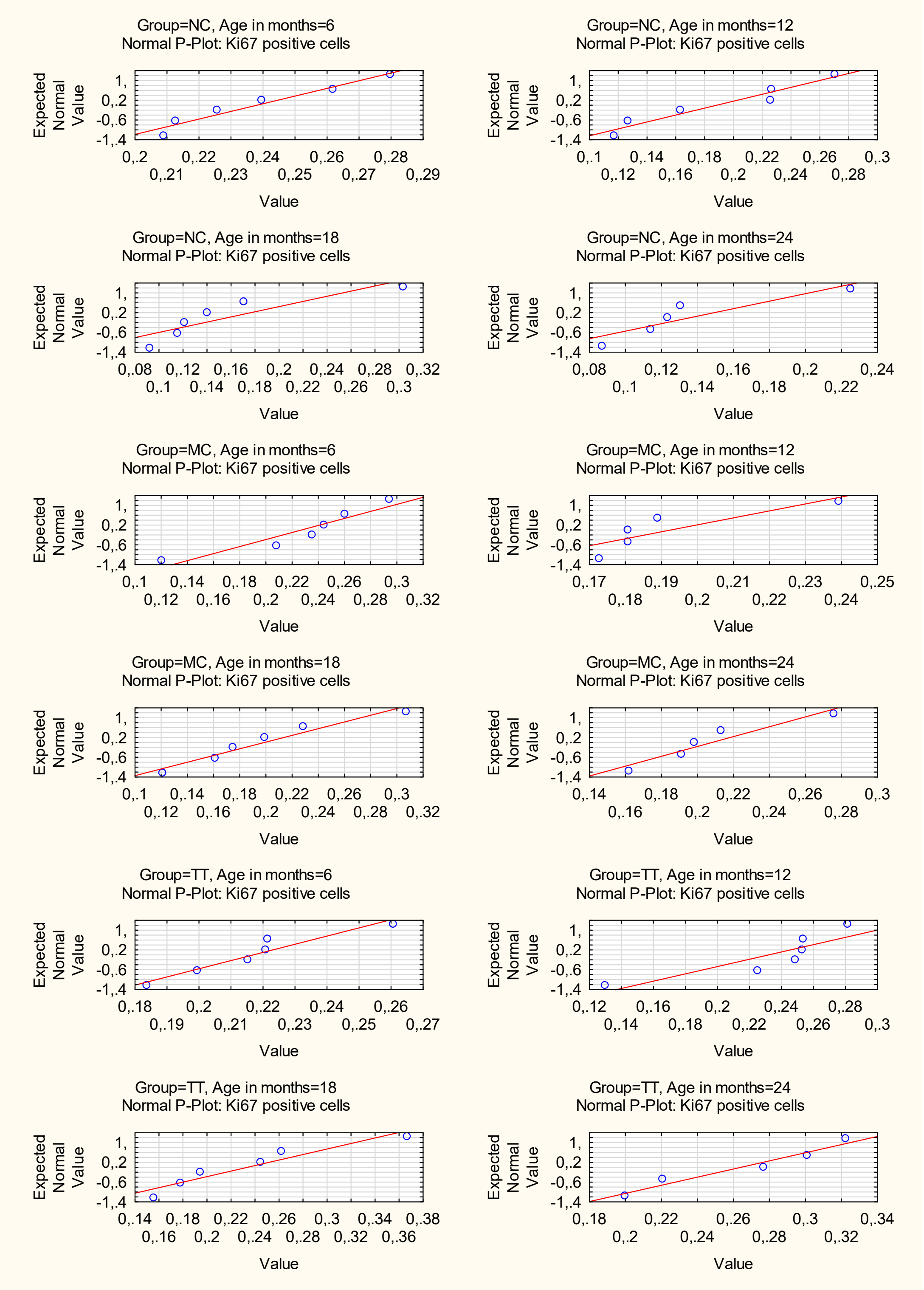

Normality test and normal probability plots suggest that we cannot reject null-hypothesis about normality of Percentage of Ki67 positive cells in all subsamples at 5% level of significance except

for group MC with x = 12. For this subsample we cannot reject normality at level of 1% of significance. Moreover, estimates of their standard deviations suggest homogeneity of variances. By Bartlett's Chi-square test (χ^2 = 12.1566, df = 11, pv = 0.3520), and Levene's test for homogeneity of variances (F = 1.0468, dfs= (11, 56), pv = 0.4197) we cannot reject null-hypothesis that population variances of all subsamples of Y are equal. In further analysis, we will assume that variable Percentage of Ki67 positive cells has normal distribution with equal population variances in all subsamples.

For each group subsamples we assume that a linear regression model

$$Y = \beta g_0 + \beta g_1 x + \varepsilon, \quad g = \text{NC, MC, TT}$$

holds with Y (Percentage of Ki67 positive cells) as dependent variable and x (Age in months) as independent variable (regressor). In addition, we can assume that random errors ε belonging to different observations are normally distributed, independent, and have equal variances (σ^2) not only within groups but also independently of the group membership. We fitted all these models as a compound linear model with six parameters: 3 intercepts and 3 slopes (i.e. each pair of parameters represents a linear model for one group), to the data. The model seemed to be significant (F = 4.2483, dfs = (6, 62), pv = 0.0012). Moreover, since we have repeated observations of Y for each different value of the regressor, we performed lack-of-fit test. The result is that we cannot reject null-hypothesis that mean values of Y linearly depend on x within each group (F = 1.8289, dfs = (3, 62), pv = 0.1512). Table S27 is the ANOVA table.

Table S27: ANOVA table for the compound linear regression model fitting of %Ki67 positive cells

Effect	Analysis of Variance,<br>Linear regression models DV: Ki67 positive cells<br>R=.539766 R2=.291348 (Adjusted for mean)				
	Sums of Squares	df	Mean Squares	F	p-value

Regress.	0.071586	6	0.011931	4.248339	0.001204
Residual	0.174120	62	0.002808		
Total	0.245706				

Estimates of the model parameters (coefficients) are in the first part of Table S28. Slopes β_{MC1} and β_{TT1} of groups MC and TT are not different from zero significantly meaning that Y in these groups does not depend on x significantly. Therefore, we can simplify the model for these groups by putting their slopes to zero: $\beta_{MC1} = 0$ and $\beta_{TT1} = 0$. Hence, we have only constants c_{MC0} and c_{TT0} to model means of Y in groups MC and TT. In such a way reduced compound model does not differ from the starting one significantly (F = 1.1270, dfs = (2, 62), pv = 0.3306). The parameter estimates of the reduced model are presented in the second part of Table S28.

Table S28: Estimates of the parameters of the linear regression models for %Ki67 positive cells

Parameter	Estimate	Std. error	t-value	pv
β_{NC0}	0.2648	0.0269	9.8408	$< 10^{-14}$
β_{NC1}	-0.0057	0.0017	-3.3820	0.0013
β_{MC0}	0.2215	0.0273	8.1196	$< 10^{-10}$
β_{MC1}	-0.0010	0.0017	-0.5764	0.5664
β_{TT0}	0.2014	0.0269	7.4858	$< 10^{-9}$
β_{TT1}	0.0023	0.0017	1.3871	0.1704
β_{NC0}	0.2648	0.0270	9.8211	$< 10^{-14}$
β_{NC1}	-0.0057	0.0017	-3.3752	0.0013
c_{MC0}	0.2072	0.0113	18.2982	$< 10^{-26}$
c_{TT0}	0.2355	0.0111	21.2680	$< 10^{-29}$

Plot of residuals of the fitted full compound model and their normal probability plot can be found in Figure S12. The estimated linear models (full and reduced) are presented in Figure 5 in the main text all together, and in Figure S13 separately.

Figure S12: Plot of % Ki67 positive cells residuals of fitted full linear models and their normal probability plot

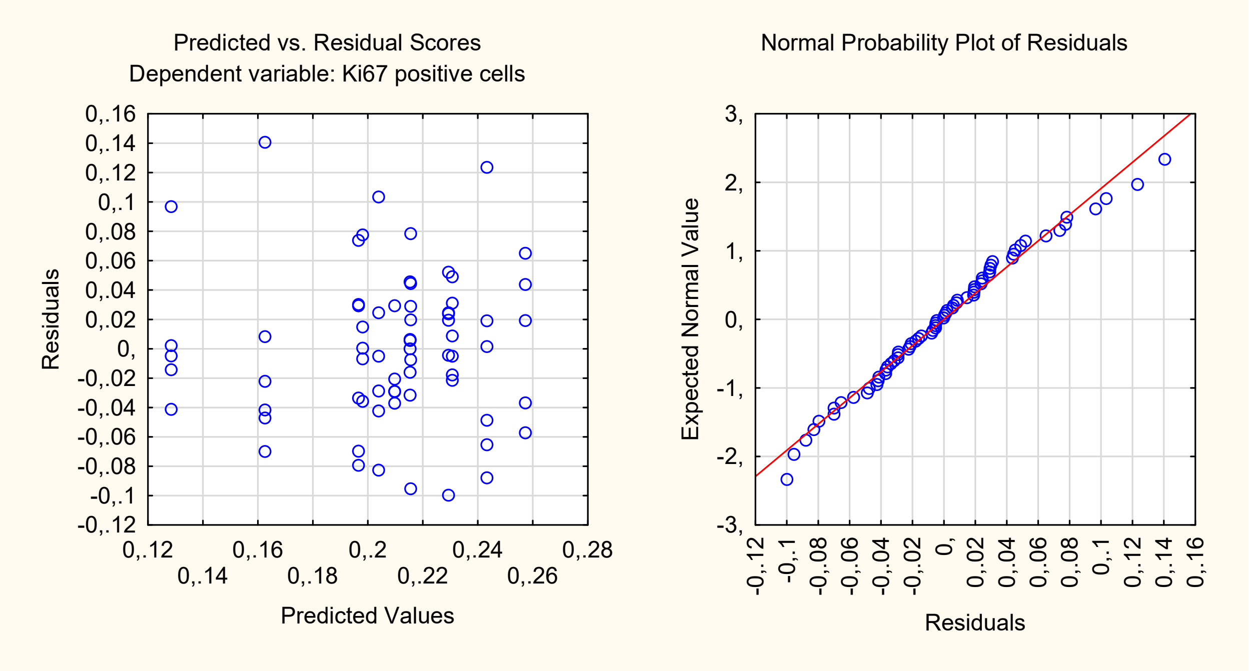

Figure S13: Mean plots of % Ki67 positive cells with error bars and their linear models by groups separately

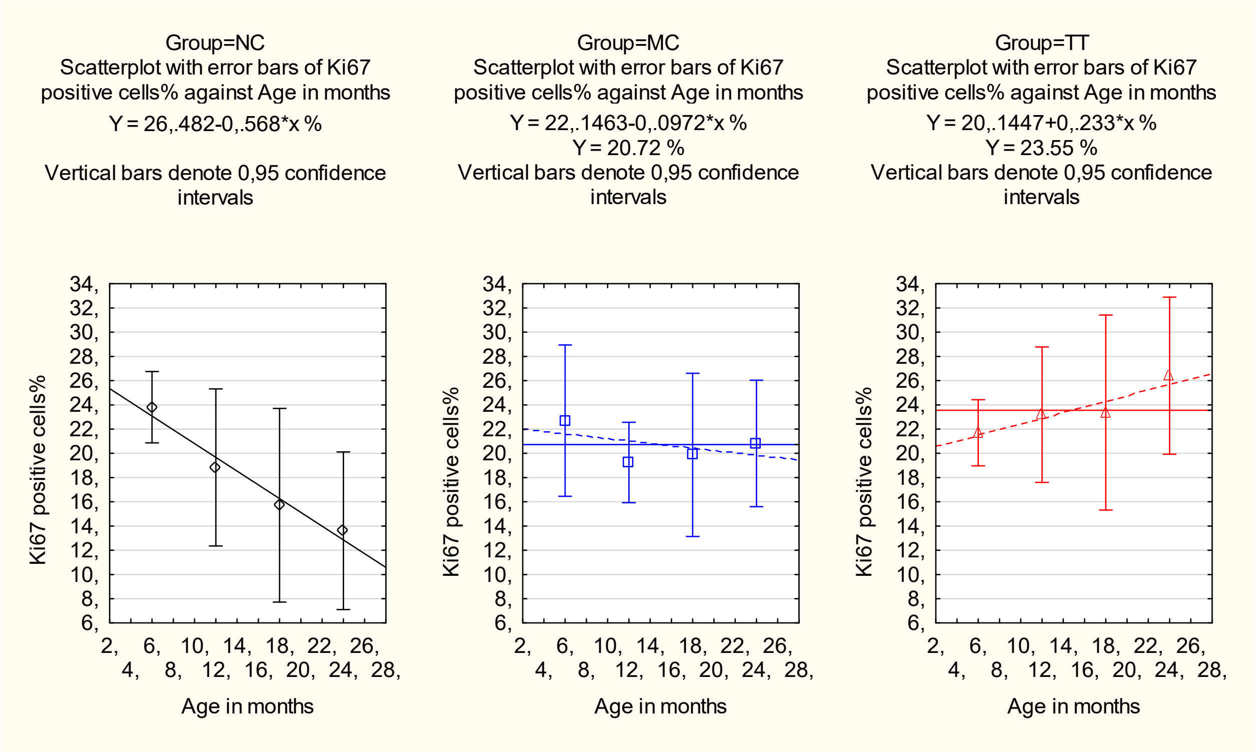

Three models: the linear model for Percentage of Ki67 positive cells against Age in months for group NC and the constant models (i.e. no dependence of %Ki67 positive cells on Age in months) for groups MC and TT, as a part of reduced compound linear model, are different significantly ($F = 7.7136$, dfs = (3, 64), pv = 0.0002). Two constant models (for groups MC and TT) are not different significantly at 5% level of significance ($F = 1.82323$, dfs = (3, 62), pv = 0.1522 when compared with respect to the full compound model, and $F = 3.2004$, dfs = (1, 64), pv = 0.0784 when compared with respect to the reduced one).

Dil labeled young neonatal skin fibroblasts

We optimized the method of transplantation using DiI labeled young neonatal skin fibroblasts isolated from isogenic Fischer 344 rats into skin of adult animals in order to determine conditions for the highest implantation efficiency. The experiments were conducted on 1year old rats. For each treatment, eight million DiI labeled cells were implanted in a 1 cm^2 skin area either one, two, or three times with a two-month interval between repeated treatments. One month following the last treatment tissue samples were collected for quantitative analysis. We measured Percentage of Dil labeled cells (% Dil Pos. Cells in tables and figures) in tissue samples one month after the last of successive (in two-months intervals) implantation of Dil labeled young neonatal skin fibroblast. The subsamples of tissues are classified with respect to total number of successive treatments: only one treatment (I.), two treatments (II.) and three treatments (III.) in total. For the purpose of statistical comparison of the groups, we took square root of the original variable values: Square-root of Dil positive cells (shortly Sqrt of Dil Pos. Cells = Y). For all data subsamples, we tested normality of Y by Lilliefors variant of Kolmogorov-Smirnov test, and by Shapiro-Wilk test. Descriptive statistics of Y together with the normality test results for the subsamples are presented in Table S29. Normal probability plots of the subsamples are in Figure S14.

Table S29: Descriptive statistics and tests of normality for Sqrt of Dil pos. cells cells

Group	No.of cases (n)	Mean ($\bar{Y}$)	Std. dev. (σ)	Std. error of mean	Lillie test L	Lillie test pv	Shapiro-Wilk test W	Shapiro-Wilk test pv
I	9	0.5668	0.1053	0.0351	0.1564	> 0.20	0.9573	0.7700
II	18	0.4588	0.0918	0.0216	0.1492	> 0.20	0.9364	0.2515
III	18	0.3513	0.0663	0.0156	0.1203	> 0.20	0.9600	0.6020

Figure S14: Normal probability plots of % Ki67 positive cells subsamples

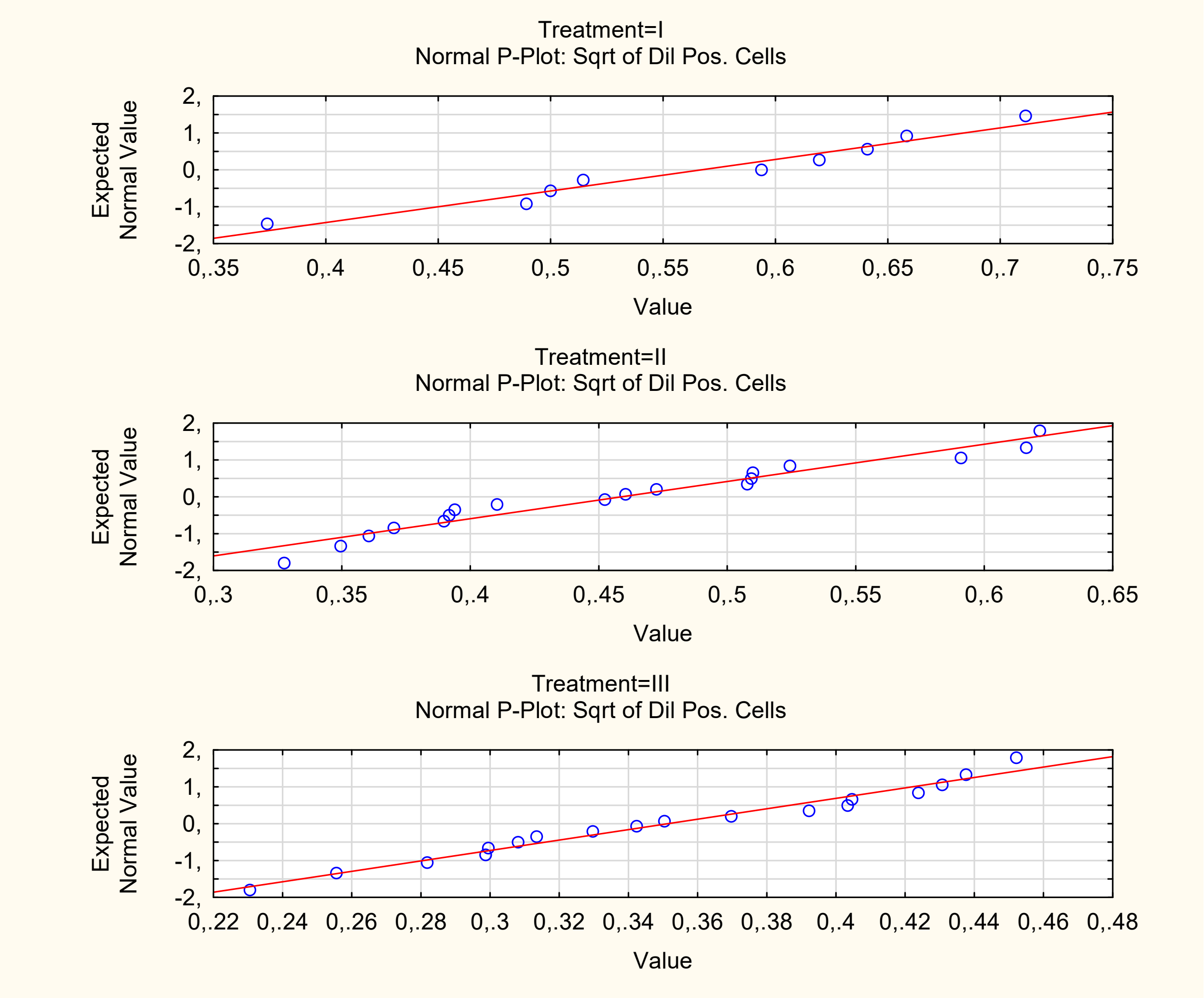

Normality test and normal probability plots suggest that we cannot reject null-hypothesis about normality of Square-root of Dil positive cells in all subsamples. Moreover, estimates of their standard deviations suggest homogeneity of variances. By Bartlett's Chi-square test ($\chi^2 = 2.7605$, df = 2, pv = 0.2515), and Levene's test for homogeneity of variances (F = 1.7737, dfs = (2, 42), pv = 0.1822) we cannot reject null-hypothesis that population variances of all subsamples of *Y* are equal. In further analysis, we will assume that variable Square-root of Dil positive cells has normal distribution with equal population variances in all subsamples.

We compared subsamples belonging to the different groups by One-Factor ANOVA method.

Table S30 is the ANOVA table.

Table S30: ANOVA table of Square-root of Dil positive cells for groups I, II, and III.

Effect	Univariate Results for Sqrt of Dil Pos. Cells; Groups: I, II, III				
	Degr. of Freedom	SS	MS	F	p-value
Treatment	2	0.2922	0.1461	20.0119	$8 \cdot 10^{-7}$
Error	42	0.3067	0.0073		
Total	44	0.5989			

We can conclude that group population means differ significantly. By applying Tuckey's HSD test and Fisher's LSD test we can say which pairs of means differ significantly. The test results are presented in Table S31: all pairs of means among groups I, II and III, differ significantly (at level 5%). Group means together with their standard error bars, and normal probability plot of the residuals are presented in Figure S15.

Table S31: Comparison of group population means of Square-root of Dil positive cells for groups I, II, and III, by Tuckey's HSD and Fisher's LSD tests.

Cell No.	Tukey HSD test; variable Sqrt of Dil Pos. Cells<br>Approximate Probabilities for Post Hoc Tests<br>Error: Between MS = .00730, df = 42.000			
	Treatment	{1}<br>.56679	{2}<br>.45883	{3}<br>.35134
1	I		0.009707	0.000119
2	II	0.009707		0.001518
3	III	0.000119	0.001518	
Cell No.	LSD test; variable Sqrt of Dil Pos. Cells<br>Probabilities for Post Hoc Tests<br>Error: Between MS = .00730, df = 42.000			
	Treatment	{1}<br>.56679	{2}<br>.45883	{3}<br>.35134
1	I		0.003499	0.000000
2	II	0.003499		0.000499
3	III	0.000000	0.000499	

Figure S15: Normal probability plot of residuals, and mean plots with error bars of Square-root of Dil positive cells

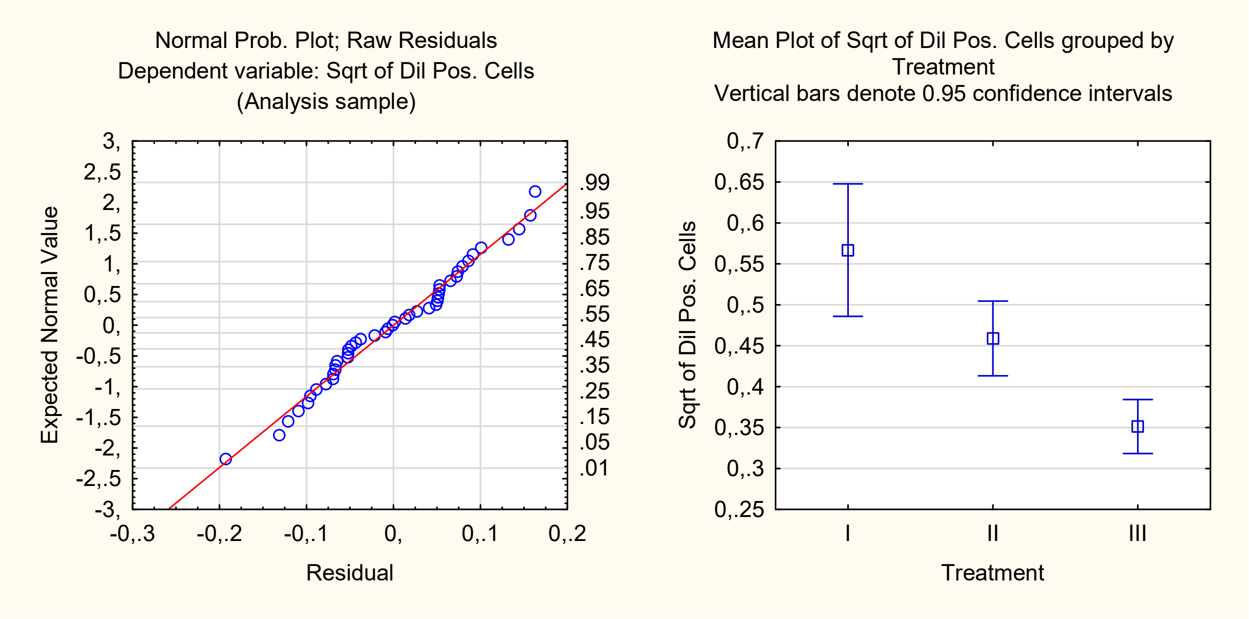

Supplement references

1. Levene, H. (1960), Robust tests for equality of variances. In Ingram Olkin; Harold Hotelling; et al. (eds.). Contributions to Probability and Statistics: Essays in Honor of Harold Hotelling. Stanford University Press, 278–292
2. Milton, J. S., and Arnold, J. C. (1987), Probability and Statistics in the Engineering and Computing Sciences, McGraw-Hill
3. Shapiro, S. S., and Wilk, M. B. (1965), An analysis of variance test for normality (complete samples), Biometrika 52, 591–611
4. Sheskin, D. J. (2000), Handbook of Parametric and Nonparametric Statistical Procedures, 2nd ed., Chapman & Hall/CRC
5. Snedecor, G. W. and Cochran, W. G. (1989), Statistical Methods, 8th ed., Iowa State University Press
